# Insights Into the Evolution of Picocyanobacteria and Phycoerythrin Genes (*mpeBA* and *cpeBA*)

**DOI:** 10.3389/fmicb.2019.00045

**Published:** 2019-01-30

**Authors:** Patricia Sánchez-Baracaldo, Giorgio Bianchini, Andrea Di Cesare, Cristiana Callieri, Nathan A. M. Chrismas

**Affiliations:** ^1^School of Geographical Sciences, University of Bristol, Bristol, United Kingdom; ^2^Institute of Ecosystem Study–Consiglio Nazionale delle Ricerche, Verbania, Italy; ^3^Department of Earth, Environment and Life Sciences, University of Genoa, Genoa, Italy; ^4^The Marine Biological Association of the United Kingdom, The Laboratory, Citadel Hill, Plymouth, United Kingdom

**Keywords:** picocyanobacteria, Synechococcus, *Cyanobium*, phycobilisomes, phylogenomics, comparative genomics, phycoerythrin

## Abstract

Marine picocyanobacteria, *Prochlorococcus* and *Synechococcus*, substantially contribute to marine primary production and have been the subject of extensive ecological and genomic studies. Little is known about their close relatives from freshwater and non-marine environments. Phylogenomic analyses (using 136 proteins) provide strong support for the monophyly of a clade of non-marine picocyanobacteria consisting of *Cyanobium, Synechococcus* and marine Sub-cluster 5.2; this clade itself is sister to marine *Synechococcus* and *Prochlorococcus*. The most basal lineage within the Syn/Pro clade, Sub-Cluster 5.3, includes marine and freshwater strains. Relaxed molecular clock (SSU, LSU) analyses show that while ancestors of the Syn/Pro clade date as far back as the end of the Pre-Cambrian, modern crown groups evolved during the Carboniferous and Triassic. Comparative genomic analyses reveal novel gene cluster arrangements involved in phycobilisome (PBS) metabolism in freshwater strains. Whilst PBS genes in marine *Synechococcus* are mostly found in one type of phycoerythrin (PE) rich gene cluster (Type III), strains from non-marine habitats, so far, appear to be more diverse both in terms of pigment content and gene arrangement, likely reflecting a wider range of habitats. Our phylogenetic analyses show that the PE genes (*mpeBA*) evolved via a duplication of the *cpeBA* genes in an ancestor of the marine and non-marine picocyanobacteria and of the symbiotic strains *Synechococcus spongiarum*. A ‘primitive’ Type III-like ancestor containing *cpeBA* and *mpeBA* had thus evolved prior to the divergence of the Syn/Pro clade and *S. spongiarum*. During the diversification of *Synechococcus* lineages, losses of *mpeBA* genes may explain the emergence of pigment cluster Types I, II, IIB, and III in both marine and non-marine habitats, with few lateral gene transfer events in specific taxa.

## Introduction

Marine picocyanobacteria, *Synechococcus* and *Prochlorococcus*, are globally distributed and make a significant contribution to primary production in open ocean waters of the subtropical and tropical regions (Partensky et al., 1999; Zwirglmaier et al., 2008; Flombaum et al., 2013). Due to their ecological significance, *Prochlorococcus* and *Synechococcus* are some of the best-known unicellular cyanobacteria with studies expanding from ecology, evolution and genomics (Hess et al., 1996; Scanlan et al., 2009; Coleman and Chisholm, 2010; Scanlan, 2012; Flombaum et al., 2013; Sánchez-Baracaldo et al., 2014; Sánchez-Baracaldo, 2015; Coutinho et al., 2016). In contrast, freshwater relatives remain poorly studied at the genomic and evolutionary levels (Cabello-Yeves et al., 2017; Di Cesare et al., 2018). Molecular ecology studies have identified small freshwater unicellular cyanobacteria as having a global distribution (Crosbie et al., 2003; Ernst et al., 2003; Sánchez-Baracaldo et al., 2008; Becker et al., 2012; Callieri et al., 2012, 2013, 2016, 2017; Fujimoto et al., 2015). In this study, picocyanobacteria are defined as all small unicellular cyanobacteria, including the free-living Syn/Pro clade (both marine and non-marine lineages) and the sponge symbiotes *Synechococcus spongiarum*. Ecologically, freshwater picocyanobacteria represent the main component of picophytoplankton in oligotrophic non-marine habitats, generally exceeding the eukaryotic fraction (Callieri, 2008; Ruber et al., 2016). In lakes and oceans picocyanobacteria play a key role in primary productivity (ranging from 5 to 80%) depending on the season, water chemistry and hydrography (Li, 1994; Callieri and Stockner, 2002; Callieri et al., 2012).

Molecular clock analyses have previously shown that marine planktonic cyanobacteria evolved toward the end of the Pre-Cambrian (Sánchez-Baracaldo et al., 2014; Sánchez-Baracaldo, 2015; Schirrmeister et al., 2015a) likely contributing to the widespread oxygenation of the oceans. Insights into the timing of the origin of marine planktonic forms have been facilitated by the increasing number of available cyanobacteria genomes. However, much less is known about the emergence of planktonic unicellar forms in non-marine habitats. It is therefore essential to determine their phylogenetic relationships to establish when non-marine picocyanobacteria first evolved. While single gene SSU rRNA phylogenies have been unable to provide enough resolution to resolve the phylogenetic relationships within picocyanobacteria, molecular ecology studies based on SSU rRNA and ITS (Sánchez-Baracaldo et al., 2008; Becker et al., 2012; Callieri et al., 2013) have revealed non-marine picocyanobacteria as a taxonomically diverse group in lakes (Sánchez-Baracaldo et al., 2008; Callieri et al., 2013). Similarly, at the genomic level, only a few studies have performed comparative genomic analyses of non-marine picocyanobacteria (Cabello-Yeves et al., 2017; Di Cesare et al., 2018). More genomic and evolutionary studies are needed in order to better understand the ecological processes underpinning the food web and biogeochemical cycles in lakes and other non-marine habitats.

Access to light is one of the main factors regulating growth and photosynthetic rates in planktonic habitats. *Synechococcus* harbors the largest pigment diversity within cyanobacteria (Six et al., 2007; Scanlan et al., 2009) allowing them to explore a wide range of light niches found in planktonic habitats including upper subsurface waters, coastal, and offshore waters in high and low latitudes (Gradinger and Lenz, 1995; Zubkov et al., 1998; Farrant et al., 2016). Phycobilisome (PBS) diversity is at the core of their ecological success. In general terms, PBS are made of a complex combination of phycobiliproteins (MacColl, 1998; Adir, 2005), in which there is a central core of allophycocyanin (APC) and six radiating phycobiliprotein-rods where phycobilin chromophores bind. Moreover, each phycobiliprotein’s α- and β-subunits may bind one to three phycobilins; these proteins have been described as phycocyanobilin (PCB: *a*_max_ ∼ 620 nm), phycoerythrobilin (PEB: *a*_max_ ∼ 545 nm) or phycourobilin (PUB: *a*_max_ ∼ 545 nm). Diversity in pigmentation is made possible by combining phycobiliproteins with various phycobilins that constitute the phycobilisome rods (Six et al., 2007). While pigment Type I strains contain the simplest rods with only phycocyanin, pigment Type II strains possess a form of phycoerythrin (PEI). In contrast, most marine *Synechococcus* have two distinct forms of phycoerythrin (PEI and PEII), which are known as pigment Type III - this has been further subdivided into four pigment subtypes according to the PUB:PEB ratio of whole cells (Six et al., 2007).

While PBS gene clusters are known to be highly conserved in marine *Synechococcus*, (Six et al., 2007; Larsson et al., 2014), the arrangement, evolution and ecological significance of the PBS clusters in non-marine *Synechococcus* has received less attention. In the genomes of most marine *Synechococcus*, there are two copies of the genes for PE (PEI and PEII)*, cpeBA* and *mpeBA* (Wilbanks et al., 1991). In this study, we traced back the evolutionary history of *cpeBA* and *mpeBA* to unravel how their evolution has contributed to pigment type diversity amongst both marine and non-marine *Synechococcus*. It is worth noting that in *Prochlorococcus* the classical cyanobacterial phycobiliproteins have been replaced; these strains have evolved a simpler pigmentation allowing them to exploit the blue light found at depth in oceanic waters (Partensky et al., 1999) which consists of chlorophyll (Chl)-derivatives divinyl-(DV) Chl (Hess et al., 1996, 2001). While *Prochlorococcus* lack phycobilisomes (Ting et al., 2002), some *Prochlorococcus* ecotypes (i.e., *Prochlorococcus* sp. SS120, *Prochlorococcus* sp. NATL2A) retain fragments of the phycobilisome in the form of a single phycobiliprotein, phycoerythrin (PE) (Hess et al., 2001). Thus, it has been inferred that the common ancestor of *Synechococcus* and *Prochlorococcus* contained phycobilins (Hess et al., 1996), yet it is unclear how these genes have evolved within the Syn/Pro clade due to the lack of genomes from non-marine strains.

To gain a deeper perspective on the evolution of non-marine picocyanobacteria genomes, we sequenced five genomes of strains isolated from lakes located in Argentina, Mexico and Europe. Phylogenomic analyses strongly support a well-defined clade containing mostly non-marine picocyanobacteria (including freshwater strains of *Synechococcus* and *Cyanobium*) and Sub-cluster 5.2, which itself is sister to the clade containing marine *Synechococcus* and *Prochlorococcus.* To gain insights into the evolution of pigments types as described by Larsson et al. (Larsson et al., 2014), we performed Bayesian phylogenetic analyses of *mpeBA* and *cpeBA* revealing that *mpeBA* originated from a duplication of *cpeBA*. The duplication event occurred prior to the divergence of *S. spongiarum* and the Syn/Pro clade. During the diversification of *Synechococcus* lineages, differences in PE gene content between pigment clusters can be attributed mostly to vertical inheritance and differential loss of these genes, with two detectable Lateral Gene Transfer (LGT) events: one between a Type III pigment cluster and *Synechococcus* sp. RCC307, the other between an ancestor of *Synechococcus* sp. CB0205 and an ancestor of *Synechococcus* sp. WH7805. Comparative analyses of newly sequenced freshwater strains show for the first time Type IIB pigment clusters previously described from metagenomes in the Baltic Sea (Larsson et al., 2014).

## Materials and Methods

### Strain Origin and Isolation

The non-marine strains studied here were isolated from lakes of different origin and limnological characteristics (Supplementary Table S1). Samples were gravity filtered through a 3 μm polycarbonate membrane and three to five replicates of 3 mL were added to 3 mL BG11 medium in small culture vial (Callieri et al., 2013). The vials were kept in a thermostat at 18–20°C and low light (10–15 μmol photons m^-2^ s^-1^). Cycloheximide (with a final concentration of 3 mmol l^-1^) was added to cultures in which picoeukaryotes were present. After a week, cultures were transferred to a vial with fresh BG11 medium. To obtain cultures with a single picocyanobacteria strain, purification was performed using flow cytometric single cell sorting with an InFlux V-GS flow cytometer (Becton Dickinson Inc.). A single cell was directly inoculated into a single well in a 96-well plate, each well was enriched with 100 μL of BG11 substrate (Callieri et al., 2013).

### DNA Extraction, Sequencing and Genome Assembly

Non-axenic cultures were grown for 4 weeks to obtain sufficient material for extraction. Biomass was centrifuged in 1.5 mL tubes, the supernatant was removed and the pellet washed in 500 μL Milli-Q water. Pellets were re-suspended in 200 μL SoluLyse (Genlantis, San Diego, CA, United States) (Hall et al., 2013) and incubated at room temperature for 15 min. Contents of the tube were transferred to a MO BIO (MO BIO Laboratories, Cambridge, United Kingdom) 0.7 mm bead beating tube and vortexed at full power for 5 min. Genomic DNA was extracted from the lysate using Machery-Nagel AXG20 (Machery-Nagel, Düren, Germany) gravity flow columns following the manufacturer’s protocol; this protocol also included the optional step of addition of lysozyme. We checked that high molecular weight gDNA was obtained by gel imaging (1% gel) and quantifying DNA concentrations using QUBIT (Invitrogen, Carlsbad, CA, United States) assay prior to sequencing. To prepare libraries, we used the Illumina Truseq Stranded Total RNA Kit (Illumina, San Diego, CA, United States) with a final average library size distribution of ∼750 bp. For sequencing we used Illumina Hi-Seq 2500 (one lane) to generate 100 bp paired-end reads with an insert size of ∼ 400 bp.

Reads were filtered based on quality and Illumina adapters were trimmed with Trimmomatic v.032 (Bolger et al., 2014) with the settings: Leading:20, Trailing:20, SlidingWindow:4:20, MinLen:50. Reads were error corrected and assembled in SPAdes v3.5.0 (Bankevich et al., 2012) using k-mer lengths of 67, 77, 87, and 97. Coverage cutoff was set to 20. Since non-axenic cultures were used to generate the genome sequences, the assembly contained contigs of non-cyanobacterial origin derived from commensal biota. To extract non-cyanobacteria sequences we used methods previously described (Chrismas et al., 2016). Assemblies were opened in the de Bruijn graph viewer Bandage (Wick et al., 2015) and BLAST databases generated for each of the assemblies. To identify cyanobacterial contigs, core cyanobacterial proteins [core CyOGs, (Mulkidjanian et al., 2006)] were BLASTed against each of these databases with tBLASTn v2.2.30+ specifying an *e*-value threshold of 1*e*-10. Connected graph nodes containing positive BLAST hits and similar read depth were retained as cyanobacteria. Unconnected nodes with either positive BLAST hits and lower read depth or similar read depth but no BLAST hits were screened independently by BLASTing against NCBI GenBank with an *e*-value threshold of 1*e*-10. To eliminate remaining non-cyanobacterial sequences, all nodes with a read depth of less than half the mean read depth of the main cyanobacterial portion of the graph were removed. Finally, all remaining contigs < 200 bp were removed to meet NCBI requirements. To determine overall coverage of each of the assemblies, reads were mapped to the assembled draft genomes using BWA (Li and Durbin, 2009).

The assembled genomes were annotated using the JGI IMG/ER pipeline (Markowitz et al., 2012) and are deposited under the following GOLD Analysis Project IDs: *Synechococcus* sp. 1G10: Gp0118737; *Synechococcus* sp. BO 8801: Gp0118738; *Synechococcus* sp. MW101C3: Gp0118739; *Vulcanococcus limneticus* LL: Gp0118741; *Synechococcus* sp. 8F6: Gp0118742. Sequences are available on NCBI GenBank under the accession numbers *Synechococcus* sp. 1G10: NQKW00000000; *Synechococcus* sp. 8F6: NQKZ00000000; *Synechococcus* sp. BO 8801: NQKY00000000; *Synechococcus* sp. MW101C3: NQKX00000000, *V. limneticus* LL: NQLA00000000.

### Taxon Sampling, Alignment and Phylogenomic Analyses

We used genomic sequence data from 131 genome taxa to study the phylogenetic relationships of the Syn/Pro clade within the context of cyanobacteria; in total 49 taxa belong to the picocyanobacteria, and 82 to other cyanobacterial groups. Genomes from non-marine picocyanobacteria were sequenced as described above, and published data were obtained from GenBank^1^. Alignments for phylogenetic analyses included a total of 136 protein-coding genes (51,865 aa); criteria explaining the genes analyzed were previously described in detail (Blank and Sánchez-Baracaldo, 2010; Sánchez-Baracaldo, 2015). Single gene alignments were generated using MAFFT v. 7 (Katoh and Standley, 2013) and concatenated using Sequence Matrix v 100.0 (Vaidya et al., 2011). ProTest v.2.4 (Abascal et al., 2005) was used to estimate the best model of evolution for the protein set. We implemented the LG model and G (gamma-distribution with four rate categories) to analyse the protein sequences. We generated a cyanobacteria phylogeny implementing Maximum likelihood, and bootstrap analyses were performed to evaluate support for branching relationships in RAxML GUI v.1.5b1 (Silvestro and Michalak, 2012).

### Bayesian Divergence Time Estimation

We applied time constraints within cyanobacteria by implementing well-recognized fossils and geochemical evidence such as the age of the Great Oxygenation Event (GOE). In this study we applied cyanobacterial fossils that have been previously implemented (Blank and Sánchez-Baracaldo, 2010; Schirrmeister et al., 2013; Sánchez-Baracaldo, 2015). For the root calibration we used the GOE, with a maximum age at 2.7 Byr (Brocks et al., 2003) and a minimum age at 2.32 Byr (Bekker et al., 2004). Within cyanobacteria we applied the following fossils: the first simple filamentous fossils of cyanobacteria *Oscillatoria* at 1.9 Byr (Hofmann, 1976); thick-walled dormant cells (akinetes) in the Nostocales at 1.6 Byr (Golubic et al., 1995); multiple fission in the Pleurocapsales at 1.7 Byr (Zhang and Golubic, 1987); a minimum age of 110 Myr for *Hemiaulus* (Sims et al., 2006) as the host of the symbiont *Richelia* (Janson et al., 1999; Foster et al., 2011); a minimum age of 100 Myr for *Braarudosphaera bigelowii* as the host for the symbiont *Atelocyanobacterium thalassa* UCYNA (Burnett, 1998; Cornejo-Castillo et al., 2016); and a maximum age of 713 Myr for as the host of the symbiont *Synechococcus spongiarum* (Love et al., 2009).

**FIGURE 1:**
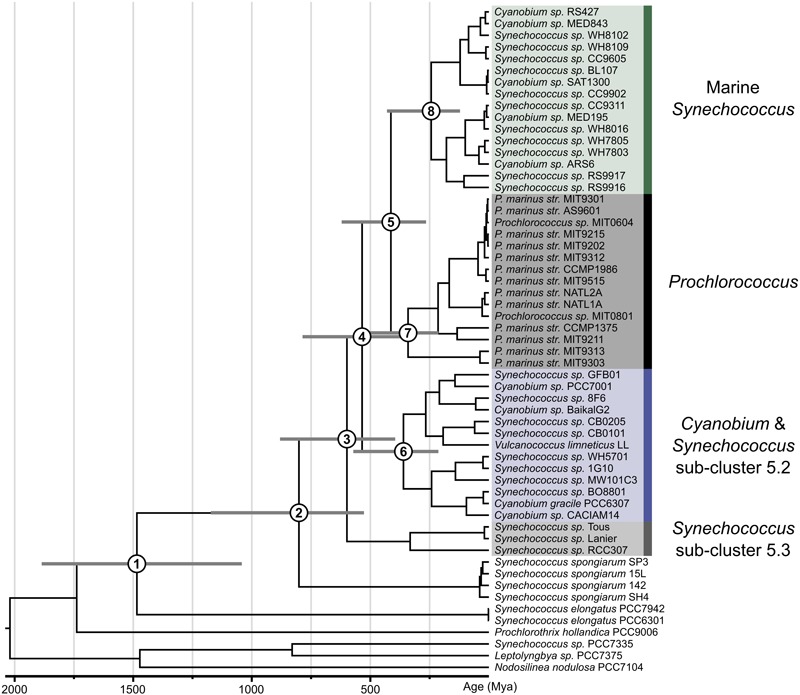
Time calibrated phylogeny of picocyanobacteria as inferred from geological time. The phylogenetic tree shown was estimated based on 136 gene. Bayesian relaxed molecular clock analyses were carried out in Phylobayes 4.1 (Lartillot et al., 2009) implementing the UGAM (42) and the CAT-GTR-G substitution model. Age estimates for the numbered nodes (1–8) indicated are given in Table 1, which includes the corresponding values for the posterior 95% confidence intervals.

Ages were estimated for the cyanobacteria topology generated by the ML analyses described above using LSU and SSU. All analyses applied seven calibration points with the aim of estimating the age divergences for the picocyanobacteria as shown in Figure 1. Divergence times were estimated under uncorrelated gamma multipliers (UGAM) (Drummond et al., 2006) in Phylobayes 4.1 (Lartillot et al., 2009). Substitutions were modeled using the CAT-GTR+G replacement model. We used a birth-death prior on divergence times, and soft bounded calibrations for calibrated nodes, which allow 5% of the prior density to fall outside the minimum–maximum interval of each calibration. Analyses were performed using a root prior in which a Gamma distribution makes 95% of the prior distribution fall between 2.32–2.7 Bya. The software Tracecomp (in Phylobayes) was used to test for convergence of molecular clock analyses.

### Comparative Genomics

Comparative analyses were performed using tools available in the JGI IMG/ER online portal. PBS gene clusters were located using BLAST searches (*e*-value threshold = 1*e*-10) using *rpcA* (SynWH7803_0479) and *cpeA* (SynWH7803_0486) as search queries. Where genomes were not available on JGI IMG/ER, genes were identified manually using BLAST. In total, genome characteristics (genome size and GC content) were obtained for 59 picocyanobacterial genomes (Supplementary Table S2). Out of these, six (*Cyanobium* sp. MED195, *Cyanobium* sp. MED843, *Cyanobium* sp. SAT1300, *Cyanobium* sp. ARS6, *Cyanobium* sp. Baikal-G2, and *Synechococcus* sp. CB0101) had incomplete or missing PBS clusters, while the full genome for NS01 could not be found publicly available. Plots were generated in R using ggplot2 and genoPlotR (Guy et al., 2010) and further edited in Inkscape^2^. Statistics were carried out using the Kruskall test function in R.

### Phylogenetic Analyses of *cpeBA* and *mpeBA*

#### Identification of *cpeBA* and *mpeBA* Orthologs

Phylogenetic relationships of *cpeBA* and *mpeBA* were based on data from 131 cyanobacteria proteomes, which were sampled using BLASTP 2.2.28+. We used query sequences *cpeA* and *cpeB* from *Synechococcus* sp. WH7805 and *mpeA* and *mpeB* from *Synechococcus* sp. BL107, with an *e*-value threshold of 10-5. Matching sequences were retrieved and later aligned using MAFFT v. 7 (Katoh and Standley, 2013). Neighbor-joining trees were constructed using *rapidnj* v. 2.3.2 (Simonsen et al., 2008). Percentage identity and alignment length of the BLAST matches were multiplied together and the resulting number of identical amino acids, expressed as a fraction of the query sequence length, was associated to each tip in the tree. Contours were drawn on the neighbor-joining tree, delimiting groups of tips with the same degree of similarity to the query sequence (Supplementary Figure S1). “True” ortholog sequences were defined as those belonging to a (mostly) monophyletic clade that contains the query sequence and has a well-defined contour profile (blue-labeled tips in Supplementary Figure S1). Sequences that fell on the boundary of the group and whose identity could not be definitively confirmed were marked as uncertain (red-labeled tips; Supplementary Figure S1) and excluded from subsequent phylogenetic analyses (if a genome had the *mpeA* gene in an uncertain position, its *mpeB* gene was also excluded).

#### Phylogenetic Analyses of Orthologs

**FIGURE 2:**
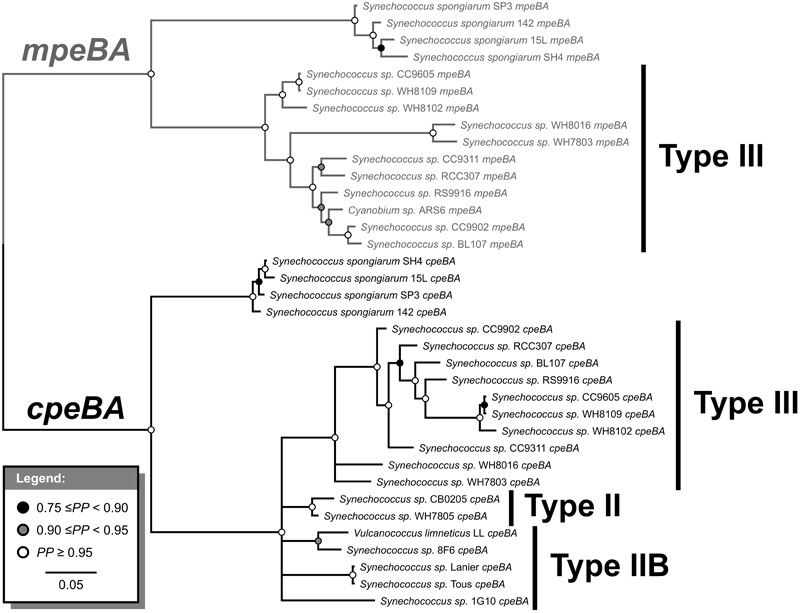
Bayesian phylogenetic tree of *cpeBA* and *mpeBA*. *PP*, posterior probability. Nodes with *PP* < 0.75 were collapsed. Pigment cluster types are highlighted; only species with a Type III pigment cluster (and *Synechococcus spongiarum*) have *mpeBA* genes.

Orthologs for each of *cpeA, cpeB, mpeA*, and *mpeB* were aligned using MAFFT v. 7 (Katoh and Standley, 2013). A Bayesian phylogenetic tree was built using MrBayes v. 3.2.6 (Ronquist et al., 2012), with a mixed amino acid model prior, invariant sites and gamma distributed site rates, run for 10,000,000 generations. Topology convergence was assessed using the ASDSF statistic in MrBayes and convergence of parameters was assessed using Tracer version 1.6 (Rambaut et al., 2013). Since previous studies have suggested that *cpeB* and *cpeA* originated via an ancient gene duplication event (Wilbanks et al., 1991; Wilbanks and Glazer, 1993), the resulting topology was rooted in the branch connecting *cpeA* and *mpeA* with *cpeB* and *mpeB* (Supplementary Figure S2). We found lack of phylogenetic signal for single-gene phylogenies. Resolution was improved by separately aligning *cpeA* with *mpeA* and *cpeB* with *mpeB* and concatenating the two alignments. We then used the concatenated alignment to build another Bayesian phylogenetic tree using MrBayes. We performed a partitioned analysis, with the *cpeA/mpeA* sequences in one partition and the *cpeB/mpeB* genes in the other partition, with all parameters unlinked except the topology and the branch length, and the same model as before (Supplementary Figure S3). We also performed a similar analysis, using the same alignments as the last one, but only including species from the Syn/Pro clade and *S. spongiarum*, under the same model and parameters as before (Figure 2).

#### Bayesian Hypothesis Testing

**FIGURE 3:**
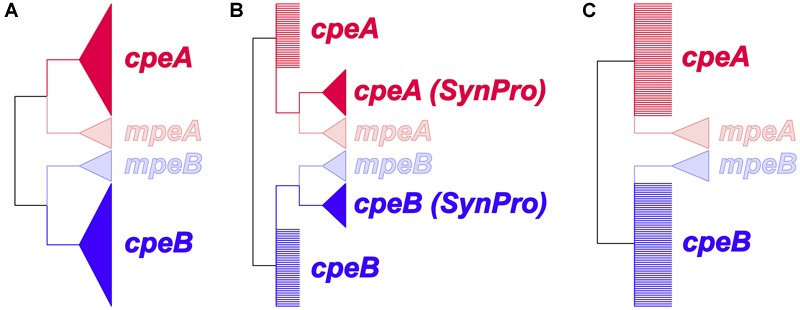
Prior constraints for the hypotheses to identify the evolutionary mechanism leading to the divergence of *mpeA* and *mpeB* from *cpeA* and *cpeB*. Wedges represent monophyletic groups; polytomies are represented as multiple branches arising from a single node. **(A)** Duplication or lateral gene transfer (LGT) before the last common ancestor of Cyanobacteria. *cpeA* and *cpeB* are constrained to form a monophyletic clade excluding, respectively, *mpeA* or *mpeB*. **(B)** Duplication in a common ancestor prior to the diversification of *S. spongiarum* and of picocyanobacterial lineages. The *cpeA (Syn/Pro)* and *cpeB (Syn/Pro)* clades contain the sequences for the respective gene, from all the picocyanobacteria (including *S. spongiarum*). **(C)** Lateral gene transfer between a crown group cyanobacterium and a common ancestor of *S. spongiarum* and the other *Synechococcus* species that possess *mpeBA* genes. This hypothesis, unlike **(A)**, does not constrain *cpeA* and *cpeB* sequences to form monophyletic clades excluding, respectively, *mpeA* or *mpeB*.

To identify the evolutionary mechanism leading to the divergence of *mpeA* and *mpeB* from *cpeA* and *cpeB*, we tested the three hypotheses shown in Figure 3. Since in unrooted phylogenies are indistinguishable, we tested the hypotheses shown in Figure 3 using the alignment of *cpeA, cpeB, mpeA*, and *mpeB* that had been used to generate the topology shown in Supplementary Figure S2. We estimated the marginal likelihood for each hypothesis using the stepping-stone algorithm (Xie et al., 2011) as implemented in MrBayes, imposing the relevant topology constraint to test each hypothesis. We used these marginal likelihoods to compute Bayes factors that quantify the relative evidence in favor of each hypothesis (Kass, 2012). Finally, we also ran similar analyses to determine whether LGT has occurred for *mpeBA* and/or *cpeBA* genes within picocyanobacteria (see Supplementary Information).

### Phylogenetic Analyses of Pigment Clusters

To verify our hypothesis about the evolution of phycobilisome pigment clusters in picocyanobacteria, we retrieved a subset of the genes shown in the clusters of Figure 4 of Larsson et al. (2014); the subset here includes 16 genes: *aplA, cpcA, cpcB, cpcG2, cpeA, cpeB, cpeC, cpeS, cpeT, cpeY, cpeZ, mpeA, mpeB, mpeV, pebA*, and *pebB*, for 51 picocyanobacterial species. We identified true ortholog sequences; from these analyses we aligned sequences for each gene using MAFFT v7.123b (Katoh and Standley, 2013). Species for which there were less than three genes out of 16 were excluded (a total of 11 were removed). With the remaining sequences, we concatenated the alignments, and built a phylogenetic tree using MrBayes v. 3.2.6 (Ronquist et al., 2012). We performed a partitioned analysis (all parameters unlinked, except tree topology and branch lengths, which were linked through a variable rate multiplier) with a mixed amino acid model, invariant sites and gamma distributed site rates, run for 10,000,000 generations.

### Spectroscopic Measurements

*In vivo* absorbance spectra of the diluted cultures were measured with a double monochromator spectrophotometer (SAFAS UVMC2) in the wavelength range 400–750 nm. Spectra were recorded with a 1 nm interval and 5 nm slit width in a quartz cuvette of 1 cm. 50 μL of NaClO 10% was directly added in the cuvette and the samples read again to record the scatter. Absorbance due to scatter was subtracted from the spectra values at each wavelength for each sample. For fluorescence spectroscopy an Horiba Scientific Fluoromax4 spectrofluorometer was used; excitation and emission slits were set at 2 nm bandpass to record the excitation spectra with emission at 580 nm in order to measure the PUB to PEB fluorescence excitation ratio (Six et al., 2007).

## Results

### Phylogenetic Analyses

Our large-scale maximum likelihood phylogenomic analyses confirm that *Synechococcus, Prochlorococcus*, and *Cyanobium* belong to a well-supported monophyletic clade (Supplementary Figure S4, 100% bootstrap support) with the freshwater *S. elongatus* strains (*S. elongatus* PCC6301, *S. elongatus* PCC7942) appearing as a sister group (100% bootstrap support). Syn/Pro and *S. elongatus* form a clade (100% bootstrap support), which is sister to the filamentous *Prochlorothrix hollandica* PCC 9006 (100% bootstrap support). All of these together are nested within the *Lyngbya*-*Phormidium*-*Plectonema* (LPP) clade (Figure 1, 100% bootstrap support). While *S. elongatus* is sister to the picocyanobacteria (node 1), these lineages diverged during the mesoproterozoic at around 1,484 Mya (95% HPD: 1,886–1,042). Our analyses strongly support (100% bootstrap support) the symbiont lineage *S. spongiarum* (*S. spongiarum* SH4, *S. spongiarum* 15L, *S. spongiarum* SP3, and *S. spongiarum* 142) as the sister group to the picocyanobacteria clade that contains both freshwater and marine strains (Burgsdorf et al., 2015). Our molecular clock analyses suggest that there is a lag between the divergence of freshwater *S. elongatus* and the emergence of a lineage leading to the Syn/Pro clade and *S. spongiarum* (node 2) toward the end of the Neoproterozoic (1,000–542 Mya) around 801 Mya (95% HPD: 1,173–527) consistent with previous relaxed molecular clock studies.

The recently sequenced freshwater strains *Synechococcus* sp. Tous and *Synechococcus* sp. Lanier, recovered from metagenomic data, form a monophyletic group with marine *Synechococcus* sp. RCC307 (Sub-cluster 5.3, 100% bootstrap support). So far this is the most basal lineage (100% bootstrap support) within the Syn/Pro clade diverging from the main picocyanobacteria clades (node 3) at around 599 Mya (95% HPD: 881–395). Newly sequenced genomes in this study reveal a well-supported (100% bootstrap support) picocyanobacteria sub-clade, mostly containing freshwater strains, a few marine (i.e., Sub-cluster 5.2) and alkaline strains, that is sister to marine *Synechococcus* (including previously described clusters I–VIII, and IX) and *Prochlorococcus* (Figure 1 and Supplementary Figure S4). This sub-clade of mostly non-marine picocyanobacteria diverged from marine picocyanobacteria (*Synechococcus* and *Prochlorococcus*) at the beginning of the Cambrian (node 4: 535 Mya with 95% HPD 786–355). The already recognized monophyletic group consisting of marine *Synechococcus* and *Prochlorococcus* (node 5) shared a common ancestor during the Devonian at around 413 Mya (95% HPD: 621–265). Crown groups of modern picocyanobacteria appeared more recently (Figure 1). Non-marine *Cyanobium* and *Synechococcus* (node 6) as well as marine *Prochlorococcus* (node 7) originated during the Carboniferous at around 360 Mya (95% HPD: 572–213) and 341 Mya (95% HPD: 528–213), respectively. Meantime the crown group of *Synechococcus* (node 8) evolved during the Triassic at around 243 Mya (430–122). Results from the relaxed molecular clock analyses are summarized in Table 1.

**Table 1:** Posterior age estimates in Myr using a Bayesian approach.

Node	Clade	Age estimate (Mya)	Eon
1	*Syn/Pro* + *Synechococcus spongiarum* + *Synechococcus elongatus*	1,484 (1,886–1,042)	Mesoproterozoic
2	*Syn/Pro* + *Synechococcus spongiarum*	801 (1,173–527)	Neoproterozoic
3	*Syn/Pro*	599 (881–395)	Neoproterozoic
4	*Cyanobium* and *Synechococcus* + *Prochlorococcus* + Marine *Synechococcus*	535 (786–355)	Phanerozoic/Cambrian
5	*Prochlorococcus* + Marine *Synechococcus*	413 (621–265)	Phanerozoic/Devonian
6	*Cyanobium* and *Synechococcus*	360 (572–213)	Phanerozoic/Carboniferous
7	*Prochlorococcus*	341 (528–213)	Phanerozoic/ Carboniferous
8	Marine *Synechococcus*	243 (430–122)	Phanerozoic/Triassic

*Node ID corresponds to those shown in Figure 1. Age estimates are given for analyses under UGAM clock model for the topology generated in RaxML. The CAT-GTR replacement model was implemented and the root was set with a maximum age of 2.7 Bya (Brocks et al., 2003) and a minimum age of 2.32 Bya (Bekker et al., 2004)*.

### Genome Traits and Structure

**FIGURE 4:**
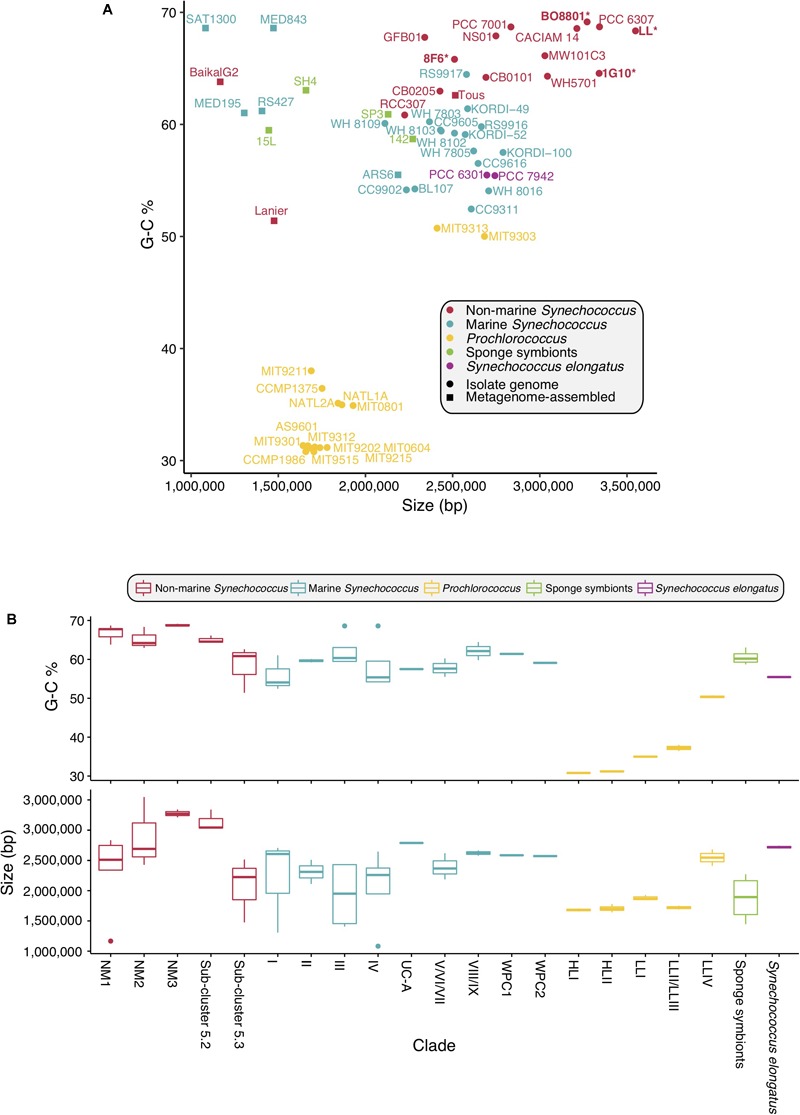
Genome size and GC content of picocyanobacteria. **(A)** GC content plotted against size. Newly sequenced genomes are in bold marked with ^∗^. **(B)** GC content and genome size within each Sub-cluster.

The genome size of picocyanobacteria within the non-marine clades varied in a broad range of values (1.2–3.5 Mbp). Non-marine picocyanobacteria strains overall tended to be larger than those from the marine *Synechococcus* clade (1.1–2.9 Mbp), *Prochlorococcus* (1.6–2.7 Mbp) and the sponge symbionts (1.4–2.3 Mbp) (Figure 4 and Supplementary Table S2). The overall range of GC content was similar between the non-marine (51.4–69.15%) and marine clades (52.45–68.6%). However, *Synechococcus* sp. Lanier was a clear outlier amongst the non-marine strains at 51.4%; all others had GC content >60%. GC content for *Prochlorococcus* was typically lower (30.79–50.74%) and ranged from 55.43–55.48% in *S. elongatus* and 58.7–63.05% in the sponge symbionts (Figure 4 and Supplementary Table S2). Within the non-marine clade, picocyanobacteria belonging to Sub-cluster 5.3 (containing *Synechococcus* sp. Tous, *Synechococcus* sp. Lanier, and *Synechococcus* sp. RCC307) had significantly lower genome sizes [χ^2^(1) = 3.5714, *p* < 0.05] and GC content [χ^2^(1) = 7, *p* < 0.05] than other members of this clade (Figure 4 and Supplementary Table S2). It is important to note that several of the smaller genomes throughout the picocyanobacteria (*Synechococcus* sp. MED195, *Synechococcus* sp. MED843, *Synechococcus* sp. SAT1300, and *Synechococcus* sp. Baikal-G2) also lacked complete PBS clusters. Since these genomes (in addition to *Synechococcus* sp. Lanier, *Synechococcus* sp. Tous, *Synechococcus* sp. RS427, *Synechococcus* sp. ARS6 *S. spongiarum* SH4, *S. spongiarum* 15L, *S. spongiarum* SP3, and *S. spongiarum* 142) were assembled from metagenomes it is possible that genome size is an underestimation for these strains (Figure 4 and Supplementary Table S2).

### PBS Subunits and Spatial Distribution

**FIGURE 5:**
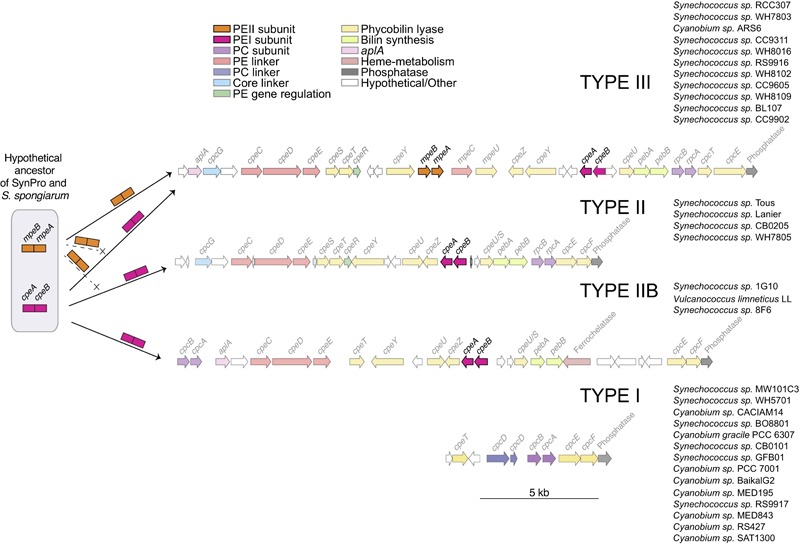
A schematic diagram showing a hypothetical ancestor of the *S. spongiarum* and the Syn/Pro clade, distribution of *cpeBA* and *mpeBA*, and spatial distribution of PBS genes. The potential distribution of *cpeBA* and *mpeBA* was inferred by phylogenetic analyses shown in Figure 2 and comparative genomic analyses shown in Figure 4 and described in the text.

General structures of the PBS clusters are shown in Figure 5. Typically, all PE containing clusters (Types III, II, and IIB) contain *cpeBA*, the phycoerythrin linker operon *cpeCDE*, phycourobilin *pebBA*, and the bilin lyases responsible for attachment of chromophores (Biswas et al., 2011) *cpeZ, cpeY* (two copies in Type III), *cpeU* (two copies in Types II and IIB) and *cpcEF* (which may be fused in Type III). Types II and III have the lyases *cpeSTR* whereas Type IIB only has *cpeT*. *cpeR* has been implicated in expression of *cpeBA* with the onset of green light (Cobley et al., 2002). Only Type III clusters contain *mpeBA* and its associated linkers *mpeC* and *mpeU*. Both Type II and III have *rpcBA* downstream of *pebBA*, where instead a ferrochelatase is found in Type IIB on the opposite strand. Instead, Type IIB has a *cpcBA* upstream of the allophycocyanin-like gene *aplA* (absent in Type II) (Montgomery et al., 2004).

Only one strain in the non-marine clade (*Synechococcus* sp. CB0205) had a PC/PE Type II cluster. We also identified the pigment cluster Type IIB (Figure 5) in non-marine picocyanobacteria genomes (including three new to this study: *Synechococcus* sp. 8F6, *V. limneticus* LL, *Synechococcus* sp. 1G10); this type of pigment cluster which had been recently reported in metagenomic analyses from low-salinity and brackish areas of the Baltic sea (Larsson et al., 2014). There were no examples of Type III clusters within the non-marine picocyanobacteria.

### Evolution of *cpeBA* and *mpeBA*

Phycoerythrin genes, *cpeBA* and *mpeBA*, shared a common ancestor prior to the split between the Syn/Pro clade and *S. spongiarum* (Figure 2); our Bayes Factor analyses show overwhelming support for the hypothesis that these genes evolved as the result of a gene duplication in this ancestor (hypothesis B in Figure 3, log-marginal likelihood: -10724.83, Supplementary Table S3), rather than a more ancient duplication (log-Bayes Factor: 34.07) or a lateral gene transfer (log-Bayes Factor: 38.83). The current distribution of *mpeBA* genes can be explained mostly by the retention of *mpeBA* by marine picocyanobacterial lineages exhibiting pigment cluster Type III (Figure 5) and loss of *mpeBA* in lineages of freshwater and brackish strains exhibiting pigment clusters Type I, Type II, and Type IIB (Figure 5). A lateral gene transfer event is likely responsible for the presence of *mpeBA* in *Synechococcus* sp. RCC307; other LGT events also likely happened, involving *Synechococcus* sp. CC9311, *Synechococcus* sp. RS9916, *Synechococcus* sp. CC9902, *Synechococcus* sp. BL107, and *Cyanobium* sp. ARS6 (see Supplementary Information).

The current distribution of *cpeBA* genes can also be explained mostly by the retention of *cpeBA* in cyanobacterial lineages exhibiting pigment clusters Type II, Type IIB, and Type III (Figure 5) and loss of *cpeBA* in lineages exhibiting pigment cluster Type I, with some *Prochlorococcus* strains losing both *cpeA* and *cpeB*, some retaining a highly derived version of both genes, and others only losing *cpeA*. *Cyanobium* sp. ARS6 appears to have lost *cpeB*, although this could be the result of an incomplete metagenome assembled genome. A LGT event appears to have again involved *Synechococcus* sp. RCC307 and a Type III pigment cluster (it is likely that a single event resulted in the LGT of the whole pigment cluster); another LGT event likely involved ancestors of *Synechococcus* sp. CB0205 and *Synechococcus* sp. WH7805 (Supplementary Information and Figure 2).

### Evolution of Pigment Clusters

The phylogenetic tree of the phycobilisome pigment clusters is shown in Supplementary Figure S5. There are many differences between this tree and the species tree (Figure 1 and Supplementary Figure S4), which might be explained either by the occurrence of many lateral gene transfer events, or by uncertainties in the tree reconstructions. Notable features of this tree are that (1) *Synechococcus* sp. RCC307 clusters with the other Type III pigment cluster species, rather than in the position expected from the species phylogeny; (2) *Synechococcus* sp. WH7805 and *Synechococcus* sp. CB0205 are sister taxa; (3) Types II and IIB pigment clusters do not form a monophyletic group.

### Pigment Characteristics

**FIGURE 6:**
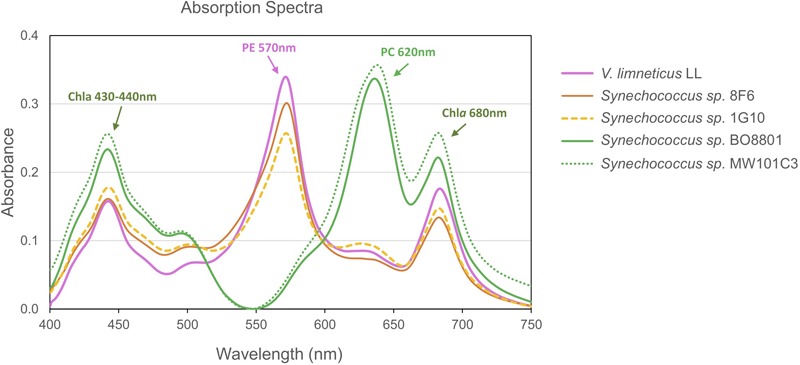
Diversity of pigment absorbance spectra of the five non-marine *Synechococcus* strains studied.

By performing the absorption spectra of PE strains, we show that strains *V. limneticus* LL, *Synechococcus* sp. 8F6, and *Synechococcus* sp. 1G10 peaked at 570 nm while the PC strains peaked at 620 nm (Figure 6). In all the strains, the chlorophyll *a* peaks (680 and 435 nm) are also clearly visible. The spectral excitation signature of our PE strains is associated with PEI (Ong and Glazer, 1991) which in general lacks PUB and has mostly PEB chromophores (Wood et al., 1985). Nevertheless, one of our strains, *Synechococcus* sp. 1G10 isolated from an ultraoligotrophic lake with blue dominant underwater radiation, showed a fluorescence excitation ratio (*F*_495_:*F*_550_) with emission at 580 nm of 0.6, indicating low PUB (Six et al., 2007). However, there was no evidence for the PUB binding linker *mpeC* (Wilbanks and Glazer, 1993; Six et al., 2005) within the genome of *Synechococcus* sp. 1G10. The other two strains (*V. limneticus* LL and *Synechococcus* sp. 8F6) showed a *F*_495_:*F*_550_ ratio of 0.3 and 0.2, respectively, indicating the absence of PUB. In strains containing the phycocyanin-only Type I PBS pigment clusters (*Synechococcus* sp. MW101C3, *Synechococcus* sp. BO8801) absorption spectra peaked at 620 nm (Figure 5).

## Discussion

### Evolution and Deep Branching Relationships Within the Picocyanobacteria

In the context of all cyanobacteria, the taxonomic diversity of free-living marine planktonic cyanobacteria is surprisingly low (Coelho et al., 2012). Marine picocyanobacteria are one of the most important phytoplankton groups due to their abundance and ubiquitous nature (Partensky et al., 1999; Johnson et al., 2006; Farrant et al., 2016). Marine *Synechococcus* and *Prochlorococcus* have been the focus of intense research, and they are relatively well understood at the ecological and genomic level. In contrast, less is known about the taxonomic diversity, genomics and evolution of their close relatives non-marine picocyanobacteria. Molecular ecology studies based on 16S rRNA and ITS genes have inferred high taxonomic diversity for non-marine picocyanobacteria (Crosbie et al., 2003; Ernst et al., 2003; Sánchez-Baracaldo et al., 2008; Becker et al., 2012; Callieri et al., 2013). However, only a handful of non-marine picocyanobacteria genomes have been sequenced (Shih et al., 2013; Cabello-Yeves et al., 2017; Di Cesare et al., 2018).

Trait evolution studies have previously shown that picocyanobacteria evolved from filamentous ancestors (Larsson et al., 2011; Schirrmeister et al., 2011; Sánchez-Baracaldo, 2015) implying that cell adhesion and intracellular communication were likely lost during the evolution of a planktonic life style (Sánchez-Baracaldo, 2015). A switch from filamentous to unicellular forms is supported by deep branching relationships within this group in which picocyanobacteria are closely related to *S. elongatus, Prochlorothrix* and the LPP clade (Figure 1 and Supplementary Figure S1). Interestingly, a similar trend was found in other cyanobacterial groups in which unicellular forms evolved from filamentous lineages in evolutionary trajectories where planktonic species are derived (e.g., *Crocosphaera* clade) (Sánchez-Baracaldo, 2015).

In geological terms, picocyanobacteria evolved relatively late toward the end of the Pre-Cambrian (Figure 1). This is consistent with the major geochemical transitions recorded in the fossil record such as changes in relative abundances of bacteria/eukaryote biomarkers (Raven, 2012; Brocks et al., 2017), and abrupt changes in geochemical proxies (Anbar and Knoll, 2002; Scott et al., 2008; Och and Shields-Zhou, 2012; Sahoo et al., 2012; Lyons et al., 2014). Understanding the timing of the origin of picocyanobacteria helps shed some light into key evolutionary events that might have contributed to shaping the carbon cycle toward the end of the Pre-Cambrian (Sánchez-Baracaldo et al., 2014). It has been widely accepted that marine *Synechococcus* and *Prochlorococcus* form a monophyletic group (Scanlan and West, 2002; Bentley and Parkhill, 2004; Flombaum et al., 2013; Batut et al., 2014; Sánchez-Baracaldo et al., 2014; Sánchez-Baracaldo, 2015); this is strongly supported by our phylogenomic analyses (Figure 1 and Supplementary Figure S4). Previous studies based on a handful of taxa have estimated *Prochlorococcus* to be ∼150 Myr old (Dufresne et al., 2005). Relaxed molecular clock analyses have significantly increased accuracy when estimating age divergence, yet taxon sampling is an additional determinant. Our age estimates for the Syn/Pro clade ∼599 Mya (Figure 1 and Table 1) are consistent with recent studies implementing a Bayesian approach showing their ancestors appearing toward the end of the Pre-Cambrian (Sánchez-Baracaldo et al., 2014; Sánchez-Baracaldo, 2015; Schirrmeister et al., 2015b).

Recently sequenced genomes, including the genomes from this study, help resolve deep-branching relationships within the picocyanobacterial shedding new light into the timing of the origin of main lineages within this clade (Figure 1 and Table 1). No doubt the inclusion of non-marine strains helps by breaking long branches, likely giving more realistic age estimates of crown groups such as marine *Synechococcus* and *Prochlorococcus.* Interestingly, the placement of early branching marine lineages such as *S. spongiarum* (Figure 1) suggests a marine origin for the Syn/Pro clade in which non-marine picocyanobacteria (*Synechococcus* and *Cyanobium*) radiated back into freshwater, brackish, halotolerant, and alkaline environments. Ancestors of the earliest divergent lineage, Sub-cluster 5.3, were likely amongst the first to colonize planktonic habitats in both freshwater and marine environments. It is worth considering that previous studies based on the sequences of 16S-23S rRNA internal transcribed spacer (ITS) (Huang et al., 2012), have shown that Sub-cluster 5.3 is far more taxonomically diverse.

The monophyletic group mostly consisting of freshwater *Synechococcus, Cyanobium* and a few marine lineages from Sub-cluster 5.2 (Figure 1) suggests that non-marine picocyanobacteria in this group shared a common ancestor with marine lineages. In this clade, there are coastal and euryhaline strains such as *Synechococcus* sp. WH5701 and *Cyanobium* PCC 7001 (Figure 1); genome analyses of these lineages suggest that these strains are capable of synthesizing Glucosylglycerol (GG) enabling them to osmoregulate in marine environments (Scanlan et al., 2009; Hagemann, 2013). It is important to emphasize that strains belonging to the non-marine *Synechococcus*/*Cyanobium* clade have been isolated from lakes from a wide range of trophic states (eutrophic to ultra-oligotrophic) and geographical regions (Supplementary Table S1) (Callieri et al., 2013). More genomic data will likely reveal a more diverse clade consisting of non-marine picocyanobacteria as suggested by previous molecular ecology studies (Crosbie et al., 2003; Sánchez-Baracaldo et al., 2008; Becker et al., 2012; Callieri, 2016). What has become clear, however, is that the major radiations of picocyanobacteria into planktonic habitats in both marine and non-marine habitats have occurred during the last 600 Mya.

### Genome Evolution and Ecology

Genome studies have pointed at a reduction in genome size (Rocap et al., 2003; Dufresne et al., 2005; Johnson et al., 2006; Luo et al., 2011) within marine *Synechococcus* and *Prochlorococcus*, with *Prochlorococcus* exhibiting the smallest genomes. Trait evolution and genomics studies have identified trends in reduction of cell diameter and genome size during the evolution of marine *Synechococcus* and *Prochlorococcus* (Scanlan et al., 2009; Larsson et al., 2011; Schirrmeister et al., 2011). Availability of new freshwater genomes expands our view on the genome sizes found within picocyanobacteria. Our study shows that the genome size of non-marine picocyanobacteria ranges from 2.5 to 3.5 Mb (Figure 4A). In contrast, genome size in marine picocyanobacteria ranges from 1.64 to 2.7 Mb in *Prochlorococcus* and from 2.2 to 2.86 Mb in *Synechococcus* (Scanlan et al., 2009) (Figure 4A). *Prochlorococcus* contains some of the smallest genomes of free-living marine cyanobacteria, and there has been strong selection for genomic streamlining shortly after the split from marine *Synechococcus* (Sun and Blanchard, 2014). Interestingly, *Prochlorococcus* has evolved in oligotrophic environments, and it is likely that natural selection favored a streamlined genome in a low-nutrient environment (Sun and Blanchard, 2014). In ultra-oligotrophic environments, genome size is constrained by small cell size which itself is a response to limited nutrient availability. Small cell sizes lead to an increased surface area:volume ratio, thus increasing nutrient uptake (Raven, 1994; Dufresne et al., 2003).

The symbiont *S. spongiarum* is widely distributed in marine sponges (Erwin et al., 2012) and it is adapted to low light (Gao et al., 2014; Burgsdorf et al., 2015). The genomes of *S. spongiarum* are smaller still, ranging from 1.4 to 1.6 Mb (Figure 4A). Genome reduction has been documented in other cyanobacteria symbionts (McCutcheon and Moran, 2011; Hilton et al., 2013; Nakayama et al., 2014; Cornejo-Castillo et al., 2016), and is consistent with comparative genomic analyses showing that the *S. spongiarum* genome has undergone streamlining in response to the sponge’s intercellular environment (Gao et al., 2014). However, it is important to remember that these are metagenome assembled genomes and potentially incomplete.

Larger genome sizes are observed in non-marine habitats, possibly due to reduced nutrient stress and the necessity to react to a greater range of environmental conditions; this is reflected in the genomes of organisms from the non-marine clade (Figure 4A). At the genomic level, most non-marine picocyanobacteria tended to be larger in size and had increased G-C content compared to marine lineages (Figure 4B). G-C content tends to be strongly correlated with genome size (Bentley and Parkhill, 2004). Since GTP and CTP are more energetically expensive, and ATP is fundamental to metabolism, small genomes tend to drift toward A-T richness (Rocha and Danchin, 2002). Furthermore, DNA repair mechanisms are commonly lost when genomes become smaller, leading to increased random mutations which tend to be in the direction of C to T and G to A, again leading to a drift toward overall A-T richness (Moran, 2002). With larger genomes comes increased G-C content, which is more metabolically expensive but may allow for higher rates of LGT (Mann and Chen, 2010), contributing to increased potential for novel gene acquisition and thus niche exploitation in organisms with larger genomes.

The exception to this apparent increased size in non-marine lineages is Sub-cluster 5.3, members of which are smaller and have lower GC content, more in keeping with that of marine strains (Figure 4B). Although the genome for *Synechococcus* sp. Lanier is incomplete, Cabello-Yeves et al. (2017) estimated it to be a similar size to *Synechococcus* sp. Tous, which is still at the lower end of the size for the clade. At present, only three genomes are available for this Sub-cluster, leading to a need to sample more of Sub-cluster 5.3.

### Evolution of Phycoerythrin Genes and Gene Clusters

In most PE-containing strains, the color and specific absorption properties of picocyanobacteria cells are mostly determined by the phycobiliproteins present in their PBS rods. Prior to the evolution of the Syn/Pro clade and *S. sporangium*, in strains such as *S. elongatus*, phycocyanin (PC) constitutes the whole rod, binding only PCB. Somewhere along the branch leading to the Syn/Pro clade and *S. sporangium* lineage, the genetic machinery to synthesize phycoerythrin (PE), as currently characterized in *Synechococcus*, evolved. Previous studies have shown that most of the PBS genes in marine *Synechococcus* are arranged into a single gene cluster, and the size of the cluster is dependent on the complexity of the rod structure (Six et al., 2007). Newly sequenced genomes of non-marine *Synechococcus* illustrate that the number of genes in pigment clusters of non-marine *Synechococcus* is variable, yet gene arrangement is highly conserved for strains possessing a particular pigment type (Figure 5; data not shown). In general terms, pigment Type III species (so far only found in marine habitats) possess more complex and larger clusters in terms of number of genes.

Comparative genomics and phylogenetic analyses shed some light into how pigments types [as described by Larsson (Larsson et al., 2014)] emerged within the picocyanobacteria. Furthermore, new non-marine picocyanobacteria genomes (*Synechococcus* sp. 8F6, *V. limneticus* LL, and *Synechococcus* sp. 1G10) reveal a novel pigment cluster, Type IIB (Figure 4), previously reported in metagenomic analyses from low-salinity and brackish areas of the Baltic sea (Larsson et al., 2014). Type IIB pigment clusters contain PEI but not PEII. Interestingly, there were no instances of Type III clusters within the non-marine picocyanobacteria (Figure 5). Genomic studies have shown that *S. spongiarum* SH4, Syn/Pro’s sister group, contains genes encoding PC, PE, and APC suggesting that this symbiont can utilize a wide spectrum of light for photosynthesis (Gao et al., 2014). In contrast, the basal and freshwater lineage, *S. elongatus* only contains genes encoding PC.

**FIGURE 7:**
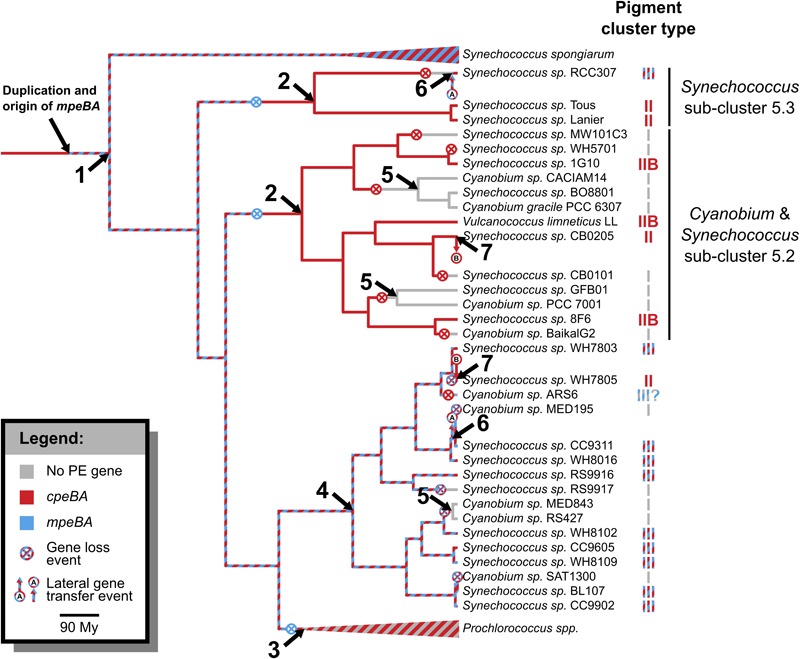
Evolution of pigment clusters in picocyanobacteria. Nodes involving significant events are: (1) The common ancestor of picocyanobacteria and *S. spongiarum* had a Type III-like pigment cluster, with both PEI and PEII genes, which were retained by *S. spongiarum* strains; (2) PEII (*mpeBA*) genes were lost in the common ancestor of Sub-cluster 5.3 and in the common ancestor of freshwater *Synechococcus* species and their close relatives, leaving them with a Type II-like pigment cluster, which diversified into polyphyletic Type II and Type IIB clusters; (3) PEII genes were also lost in the ancestor of *Prochlorococcus* strains, whose pigment clusters evolved at a very fast pace; (4) PEII genes were retained in the ancestor of marine *Synechococcus*, most of which have a Type III pigment cluster; (5) Some strains in Sub-cluster 5.2 lost their PEI genes, and some strains of marine *Synechococcus* lost both PEI and PEII, leaving all of them with a Type I pigment cluster. (6) *Synechococcus* sp. RCC307 replaced its pigment cluster (probably a Type II) with a Type III pigment cluster; and (7) *Synechococcus* sp. WH7805 replaced its pigment cluster (probably a Type III) with a Type II pigment cluster originating from an ancestor of *Synechococcus* sp. CB0205.

Phylogenetic analyses of *cpeBA* and *mpeBA* show that these two genes shared a common ancestor prior to the divergence of *S. spongiarum* and the Syn/Pro clade (Figure 2); analyses of these genes provide insights into how phycoerythrin pigment clusters Types II, IIB, and III have evolved amongst picocyanobacterial. Whilst Type III strains retained both *cpeBA* and *mpeBA*, Type IIB and Type II only retain a copy of *cpeBA* (Figure 2, 7). Moreover, variation in PE copy number in pigment cluster Types II, IIB, and III likely emerged from gene duplication, as inferred by our Bayes Factor analysis (Supplementary Table S3). This event likely occurred prior to the split between *S. spongiarum* and the Syn/Pro clade (Figure 2). Furthermore, pigment cluster Types I, II, and IIB are polyphyletic providing evidence that independent gene losses (events 2 and 5 in Figure 7) were involved in the evolution of pigment cluster types.

While gene duplication played an important role during the evolution of pigment types early on within picocyanobacteria, LGT events have also played a role more recently. Our analyses provide evidence for two major LGT events within picocyanobacteria. The first one involves the acquisition of a Type III pigment cluster by *Synechococcus* sp. RCC307, which is likely the result of the transfer of a whole pigment cluster from another marine species (Supplementary Figure S5 and Figure 7). The second LGT event involves two strains that possess a Type II pigment cluster, *Synechococcus* sp. CB0205 and *Synechococcus* sp. WH7805. A LGT event involving *Synechococcus* sp. CB0205 had been previously identified (Larsson et al., 2014). While it was previously suggested that *Synechococcus* sp. CB0205 lost its PEI genes, and re-acquired them from a marine strain though LGT, our analyses show that *Synechococcus* sp. CB0205 most likely retained *cpeBA*, and marine *Synechococcus* sp. WH7805 later acquired its Type II pigment cluster through a LGT. Furthermore, phylogenetic analyses of pigment clusters revealed additional evidence for these LGT events: *Synechococcus* sp. RCC307 clusters with other Type III species rather than with its relatives according to the species phylogeny (Supplementary Figure S4) providing evidence for a LGT event involving the whole cluster (event 6 in Figure 7), and *Synechococcus* sp. WH7805 and *Synechococcus* sp. CB0205 are sister taxa (Supplementary Figure S4), suggesting that another LGT event involving the whole cluster occurred between ancestors of these two species (event 7 in Figure 7). By combining phylogenetic analyses, comparative genomics and Bayesian statistics, our analyses have revealed the evolutionary mechanisms underpinning the evolution of phycoerythrin pigment clusters amongst the picocyanobacteria clade.

## Conclusion

All picocyanobacteria appear to have evolved from a freshwater ancestor which moved into a marine habitat between ∼1500 and ∼800 Mya; extant non-marine picocyanobacteria (such as some strains of *Synechococcus* and *Cyanobium*) have therefore moved back into freshwater environments more recently. Phycoerythrin genes and pigment clusters Types II, IIB, and III likely evolved as the result of a combination of a gene duplication, differential losses and few LGT events.

## Author Contributions

PS-B, NC, and CC designed the research. CC and ADC provided samples and characterized strains. NC extracted genomic DNA. PS-B, GB, and NC performed the phylogenomic and comparative genomic analyses. PS-B, GB, NC, ADC, and CC wrote the manuscript.

## Conflict of Interest Statement

The authors declare that the research was conducted in the absence of any commercial or financial relationships that could be construed as a potential conflict of interest.

## Footnotes

1 http://www.ncbi.nlm.nih.gov

2 http://inkscape.org
