# Supplementary material for: Insights Into the Evolution of Picocyanobacteria and Phycoerythrin Genes (mpeBA and cpeBA)
Source: Front Microbiol. 2019 Jan 30;10:45. doi: 10.3389/fmicb.2019.00045 (PMC6363710; doi:10.3389/fmicb.2019.00045)
Supplement: Supplementary file 1 [file Data_Sheet_1.docx]

**Supplementary Information**

Insights into the evolution of picocyanobacteria and phycoerythrin genes (*mpeBA* and *cpeBA*)

Patricia Sánchez-Baracaldo^1^, Giorgio Bianchini^1^, Andrea Di Cesare^2^, Cristiana Callieri^2^ and Nathan A. M. Chrismas^1,3^

^1^ School of Geographical Sciences, University of Bristol, Bristol BS8 1SS, UK

^2^ Institute of Water Research – CNR, Largo Tonolli 50, 28922 Verbania, Italy

^3^ Marine Biological Association of the United Kingdom, The Laboratory, Citadel Hill, Plymouth Pl12PB, UK

Corresponding author ([p.sanchez-baracaldo@bristol.ac.uk](mailto:p.sanchez-baracaldo@bristol.ac.uk))

*Corresponding author for this manuscript is:

Dr Patricia Sánchez-Baracaldo

Royal Society University Research Fellow

School of Geographical Sciences

University of Bristol,

Bristol, BS8 1SS

United Kingdom

Phone: +44 (0) 117 954 6858

Email: [p.sanchez-baracaldo@bristol.ac.uk](mailto:p.sanchez-baracaldo@bristol.ac.uk)

**Material and Methods**

**Lateral gene transfer assessment in the *mpeBA* phylogeny**

To assess the importance of LGT events in the evolutionary history of *mpeBA* and *cpeBA)*, we first built a phylogeny of the *mpeBA* and *cpeBA* sequences of the picocyanobacteria (using the same alignment that we used for Fig. S3, but only including the *mpeBA* and *cpeBA* sequences of picocyanobacteria). This tree was built using MrBayes v 3.2.6 , with a mixed amino acid model prior, invariant sites and gamma distributed site rates, run for 10,000,000 generations. We compared the gene phylogeny (Fig. 3) to the species/genome tree (Fig. 1), and noted that the incongruences between the species tree and the *mpeBA* tree can be explained most parsimoniously with 4 LGT events: one involving *Synechococcus sp.* RCC307 and *S. sp.* CC9311 and the other three involving *S. sp.* CC9311, *S. sp.* RS9916, *S. sp.* CC9902, *S. sp.* BL107 and *Cyanobium sp.* ARS6. Some more LGT might also have happened within the *S. spongiarum* *mpeBA* phylogeny.

We thus considered the five hypotheses represented in the topologies shown in Fig. S6. We estimated the marginal likelihood for each hypothesis using the stepping-stone algorithm implemented in MrBayes and computed Bayes factors (Table S4), only including in the analysis the *mpeBA* sequences, with *cpeBA* of *Synechococcus spongiarum* SH4 as an outgroup.

The tree shown in Fig. S6A is compatible with all five hypotheses postulated (Fig. 2). To compute accurate marginal likelihoods for these hypotheses, we used the approach described below under **Model approximations in Bayes factor analyses**. Our analyses suggest that *Synechococcus sp.* RCC307 has obtained its copy of *mpeBA* through LGT, with additional LGT events involving *S.* sp. CC9311, *S.* sp. RS9916, *S.* sp*.* CC9902, *S.* sp. BL107 and *Cyanobium* sp. ARS6. Uncertainties in the reconstruction of *S. spongiarum* *mpeBA* clusters are also present.

**Lateral gene transfer assessment in the *cpeBA* phylogeny**

To analyse LGT events in the evolutionary history of *cpeBA* in picocyanobacteria, we performed a similar approach to one mentioned above. The phylogeny of *cpeBA* (Fig. 3) exhibits low support values. We have therefore tested the four pairs of alternative hypotheses as shown in Fig. S7.

We estimated the marginal likelihoods for these hypotheses and performed Bayes factor analyses (Table S5) as described above. Here, we have used the *cpeBA* sequences and *mpeBA* of *Synechococcus spongiarum* SH4 as an outgroup (the prior used for H_1-1_ is also compatible with H_1-2_, but the approximation error is negligible. For a detail explanation see section under **Model approximations in Bayes factor analyses** below. Our results suggest that: 1) some LGT happened during the evolution of *cpeBA* in picocyanobacteria (ln-Bayes Factor: 278.43), 2) *cpeBA* of *S.* sp. CB0205 and *S. sp.* WH7805 form a monophyletic group (ln-BF: 9.55), indicating that an LGT event happened between some ancestors of these two species, 3) the direction of this LGT was from an ancestor of *S.* sp. CB0205 to an ancestor of *S.* sp. WH7805 (ln-BF: 31.95) and another LGT happened between a Type III pigment cluster species and an ancestor of *S.* sp. RCC307 (ln-BF: 33.63).

We therefore propose that the Type III pigment cluster of *Synechococcus* sp. RCC307 may have originated by a single LGT event transferring multiple genes (or even the whole cluster) from another Type III pigment cluster species. In contrast to previous hypothesis (Larsson et al 2014), Type II pigment clusters arose as the result of an LGT event from a coastal/brackish species (ancestor of *S.* sp. CB0205) to a marine species (ancestor of *S.* sp*.* WH7805).

**Model approximations in Bayes factor analyses**

The prior tree shown in Fig. 1C is not only compatible with hypothesis 3, but also with hypothesis 1 and 2 (Fig. 3). Here, we show that the marginal likelihood of hypothesis 3 can be approximated using the tree in Fig. 1C with a negligible error. The models that we tested for hypotheses 1, 2 and 3 ($H_{1}, H_{2}, H_{3}$) consist in the prior tree ($T_{1},T_{2},T_{3}$, which are different for each hypothesis) and in other parameters ($\Theta$, which are the same for every hypothesis). As stated, the tree in Fig. 1C (let it be called $T_{3}^{*}$) is not the tree that should have been used to actually test for hypothesis 3 ($T_{3}^{*}\neq T_{3}$).

The marginal likelihood of the data $D$ given the tree prior $T$ and the other model parameters $\Theta$ is $\mathbb{P}\left( D | T\cap\Theta\right)$. We are thus interested in determining $\mathbb{P(}D|T_{3}\cap\Theta)$, knowing that $T_{3}^{*}=T_{3}\cup T_{2}\cup T_{1}$ and the values of $\mathbb{P}\left( D | T_{3}^{*}\cap\Theta\right)$, $\mathbb{P}\left( D | T_{2}\cap\Theta\right)$ and $\mathbb{P}\left( D | T_{1}\cap\Theta\right)$.

We have:

$$\mathbb{P}\left( D | T_{3}^{*}\cap\Theta\right)=\frac{\mathbb{P}\left( T_{3}^{*}\cap\Theta| D \right)\mathbb{P}\left( D \right)}{\mathbb{P(}T_{3}^{*}\cap\Theta)}=\frac{\mathbb{P}\left( (T_{3}\cup T_{2}\cup T_{1})\cap\Theta| D \right)\mathbb{P}\left( D \right)}{\mathbb{P(}T_{3}^{*}\cap\Theta)}=\frac{\left( \mathbb{P}\left( T_{3}\cap\Theta| D \right)\mathbb{+P}\left( T_{2}\cap\Theta| D \right)\mathbb{+P}\left( T_{1}\cap\Theta| D \right) \right)\mathbb{P}\left( D \right)}{\mathbb{P}\left( T_{3}\cap\Theta\right)\mathbb{+P}\left( T_{2}\cap\Theta\right)\mathbb{+P(}T_{1}\cap\Theta)}=\frac{\left( \frac{\mathbb{P}\left( D | T_{3}\cap\Theta\right)\mathbb{P}\left( T_{3}\cap\Theta\right)}{\mathbb{P}\left( D \right)}+\frac{\mathbb{P}\left( D | T_{2}\cap\Theta\right)\mathbb{P}\left( T_{2}\cap\Theta\right)}{\mathbb{P}\left( D \right)}+\frac{\mathbb{P}\left( D | T_{1}\cap\Theta\right)\mathbb{P}\left( T_{1}\cap\Theta\right)}{\mathbb{P}\left( D \right)} \right)\mathbb{P}\left( D \right)}{\mathbb{P(}T_{3}^{*}\cap\Theta)}=\frac{\mathbb{P}\left( D | T_{3}\cap\Theta\right)\mathbb{P}\left( T_{3} \right)\mathbb{P}\left( \Theta\right)\mathbb{+P}\left( D | T_{2}\cap\Theta\right)\mathbb{P}\left( T_{2} \right)\mathbb{P}\left( \Theta\right)\mathbb{+P}\left( D | T_{1}\cap\Theta\right)\mathbb{P}\left( T_{1} \right)\mathbb{P}\left( \Theta\right)}{\mathbb{P}\left( T_{3}^{*} \right)\mathbb{P}\left( \Theta\right)}=\frac{\mathbb{P}\left( D | T_{3}\cap\Theta\right)\mathbb{P}\left( T_{3} \right)\mathbb{+P}\left( D | T_{2}\cap\Theta\right)\mathbb{P}\left( T_{2} \right)\mathbb{+P}\left( D | T_{1}\cap\Theta\right)\mathbb{P}\left( T_{1} \right)}{\mathbb{P}\left( T_{3}^{*} \right)}$$

Since we assume a uniform prior on the tree topologies, the prior probabilities of each tree model $T_{i}$ only depends on the number of trees that are compatible with it ($N_{i}$) and the total number of trees that are possible with the 140 sequences in our dataset ($N_{tot}$): $\mathbb{P}\left( T_{i} \right)=\frac{N_{i}}{N_{tot}}$.

Thus:

$$\mathbb{P}\left( D | T_{3}^{*}\cap\Theta\right)=\frac{\mathbb{P}\left( D | T_{3}\cap\Theta\right)N_{3}\mathbb{+P}\left( D | T_{2}\cap\Theta\right)N_{2}\mathbb{+P}\left( D | T_{1}\cap\Theta\right)N_{1}}{N_{3}^{*}}$$

$$\mathbb{P}\left( D | T_{3}\cap\Theta\right)=\frac{{\mathbb{P}\left( D | T_{3}^{*}\cap\Theta\right)N}_{3}^{*}\mathbb{-P}\left( D | T_{2}\cap\Theta\right)N_{2}\mathbb{-P}\left( D | T_{1}\cap\Theta\right)N_{1}}{N_{3}}=\frac{{\mathbb{P}\left( D | T_{3}^{*}\cap\Theta\right)N}_{3}^{*}\mathbb{-P}\left( D | T_{2}\cap\Theta\right)N_{2}\mathbb{-P}\left( D | T_{1}\cap\Theta\right)N_{1}}{N_{3}^{*}-N_{2}-N_{1}}$$

The relative error we make when approximating $\mathbb{P}\left( D | T_{3}\cap\Theta\right)$ with $\mathbb{P}\left( D | T_{3}^{*}\cap\Theta\right)$ is thus:

$$\left| \frac{\mathbb{P}\left( D | T_{3}\cap\Theta\right)\mathbb{-P}\left( D | T_{3}^{*}\cap\Theta\right)}{\mathbb{P}\left( D | T_{3}\cap\Theta\right)} \right|=\left| 1-\frac{\mathbb{P}\left( D | T_{3}^{*}\cap\Theta\right)}{\mathbb{P}\left( D | T_{3}\cap\Theta\right)} \right|=\left| 1-\frac{1}{\frac{\mathbb{P}\left( D | T_{3}\cap\Theta\right)}{\mathbb{P}\left( D | T_{3}^{*}\cap\Theta\right)}} \right|=\left| 1-\frac{1}{\frac{N_{3}^{*}-\frac{\mathbb{P}\left( D | T_{2}\cap\Theta\right)}{\mathbb{P}\left( D | T_{3}^{*}\cap\Theta\right)}N_{2}-\frac{\mathbb{P}\left( D | T_{1}\cap\Theta\right)}{\mathbb{P}\left( D | T_{3}^{*}\cap\Theta\right)}N_{1}}{N_{3}^{*}-N_{2}-N_{1}}} \right|$$

We now need to compute the $N_{i}$, knowing that for $n$ taxa there are $N_{r,n}=\frac{\left( 2n-3 \right)!}{2^{n-2}\left( n-2 \right)!}$ possible (rooted) tree topologies .

For tree $T_{1}$, we have four monophyletic groups (*cpeA*, with 52 sequences; *cpeB*, with 58 sequences; *mpeA* and *mpeB*, both with 15 sequences), which can only be arranged in one way, so the number of possible trees is the product of the number of topologies that are possible for each of these groups:

$$N_{1}=N_{r,52}\cdot N_{r,58}\cdot N_{r,15}\cdot N_{r,15}=\frac{101!\cdot113!\cdot27!\cdot27!}{2^{132}\cdot50!\cdot56!\cdot15!\cdot15!}\approx1.239\cdot{10}^{197}$$

For tree $T_{2}$, let’s consider separately *mpeA* with *cpeA* and *mpeB* with *cpeB*. The *mpeA* monophyletic group contains 15 sequences, which can only be joined in one way with the 22 sequences of the SynPro *cpeA*. These two groups together can fall in any position with regards to the other 30 *cpeA* sequences, and they thus behave as a single additional sequence. Similar reasonings are true for *mpeB* (15 sequences), SynPro *cpeB* (22 sequences) and the other *cpeB* (36 sequences). Thus:

$$N_{2}=N_{r,15}\cdot N_{r,22}\cdot N_{r,31}\cdot N_{r,15}\cdot N_{r,22}\cdot N_{r,36}=\frac{27!\cdot41!\cdot59!\cdot27!\cdot41!\cdot69!}{2^{129}\cdot13!\cdot20!\cdot29!\cdot13!\cdot20!\cdot34!}\approx7.723\cdot{10}^{168}$$

Finally, for tree $T_{3}^{*}$, we have the monophyletic groups of *mpeA* and *mpeB* (15 sequences each) that behave as a single sequence with respect to the 52 *cpeA* sequences and 58 *cpeB* sequences. Thus:

$$N_{3}^{*}=N_{r,15}\cdot N_{r,53}\cdot N_{r,15}\cdot N_{r,59}=\frac{27!\cdot103!\cdot27!\cdot115!}{2^{134}\cdot13!\cdot51!\cdot13!\cdot57!}\approx6.470\cdot{10}^{205}$$

We also know the values for the marginal likelihoods:

$$\mathbb{P}\left( D | T_{1}\cap\Theta\right)\approx e^{-10758.90}\approx{10}^{-4672.53}\mathbb{P}\left( D | T_{2}\cap\Theta\right)\approx e^{-10724.83}\approx{10}^{-4657.73}$$

$$\mathbb{P}\left( T_{3}^{*}\cap\Theta\right)\approx e^{-10763.66}\approx{10}^{-4674.60}$$

Substituting these values in the relative error formula we obtain:

$$\left| \frac{\mathbb{P}\left( D | T_{3}\cap\Theta\right)\mathbb{-P}\left( D | T_{3}^{*}\cap\Theta\right)}{\mathbb{P}\left( D | T_{3}\cap\Theta\right)} \right|\approx\left| 1-\frac{1}{\frac{6.470\cdot{10}^{205}-\frac{{10}^{-4657.73}}{{10}^{-4674.60}}7.723\cdot{10}^{168}-\frac{{10}^{-4672.53}}{{10}^{-4674.60}}1.239\cdot{10}^{197}}{6.470\cdot{10}^{205}-7.723\cdot{10}^{168}-1.239\cdot{10}^{197}}} \right|\approx2.23\cdot{10}^{-7}$$

This final computation, as well as the previous ones involving factorials, were done using Wolfram|Alpha . This result shows that approximating $\mathbb{P}\left( D | T_{3}\cap\Theta\right)$ with $\mathbb{P}\left( D | T_{3}^{*}\cap\Theta\right)$ results indeed in a very small error.

A similar analysis shows that the relative approximation error committed in the Bayes factor analysis for LGT within *mpeBA* sequences is $\approx9.38$, which is not negligible; we should therefore use a formula with analogous derivation to the previous one to compute the exact value of $\mathbb{P}\left( D | T_{1}\cap\Theta\right)$:

$$\mathbb{P}\left( D | T_{1}\cap\Theta\right)=\frac{{\mathbb{P}\left( D | T_{1}^{*}\cap\Theta\right)N}_{1}^{*}\mathbb{-P}\left( D | T_{2}\cap\Theta\right)N_{2}\mathbb{-P}\left( D | T_{3}\cap\Theta\right)N_{3}\mathbb{-P}\left( D | T_{4}\cap\Theta\right)N_{4}\mathbb{-P}\left( D | T_{5}\cap\Theta\right)N_{5}}{N_{1}^{*}-N_{2}-N_{3}-N_{4}-N_{5}}$$

Substituting the values for the marginal likelihoods (Table S4) and for the numbers of trees ($N_{1}^{*}\approx2.13\cdot{10}^{14}$, $N_{2}=1$, $N_{3}=27$, $N_{4}\approx2.29\cdot{10}^{5}$, $N_{5}\approx3.44\cdot{10}^{6}$), we obtain $\mathbb{P}\left( D | T_{1}\cap\Theta\right)\approx e^{-2973.93}$.

In the Bayes factor analyses for LGT within *cpeBA*, the prior for H_1-1_ is also compatible with H_1-2_; however, the same analysis as before shows that the relative error committed with this approximation is negligible ($\approx3.12\cdot{10}^{-24}$).

**Tables**

**Table S1.** Characteristics of the lake of origin for the strains isolated and sequenced.

| Strain | LL | BO8801 | MW101C3 | 1G10 | 8F6 |
| --- | --- | --- | --- | --- | --- |
| Lake of origin | Albano | Constance | Mondsee | Nahuel Huapi | Alchichica |
| Depth of origin (m) | 0.5 | - | - | 70 | 2 |
| Latitude N | 41°45’ | 47°39’ | 47°49’ | 40°26’S | 19°24’ |
| Longitude E | 12°40’ | 9°18’ | 13°24’ | 71°33’W | 97°24’ |
| Altitude (m a.s.l.) | 291 | 395 | 481 | 764 | 2340 |
| Area (km^2^) | 6.0 | 571 | 14.2 | 557 | 2.3 |
| Maximum depth (m) | 170 | 251 | 68 | 464 | 62 |
| pH | 8.5 | 7.5 | 7.9 | 7.5 | >8 |
| Epilimnetic  Temperataure (°C) | 7.5-14 | 6.5-20 | 6.5-20 | 7-17 | 14-25 |
| Total P (µg L^-1^) | 40 | 36 | 10.4 | 4.9 | 4-16 |
| Total-N (µg L^-1^) | 400 | 900 | 600 | 67 | 12-40 DIN |
| Lake origin | volcanic | glacial | glacial | glacial | volcanic |

**Table S2.** Genome size, GC content and phylogenetic affinity for fifty-nine picocyanobacteria genomes.

| ID | GROUP | SIZE | GC | CLADE | TYPE |
| --- | --- | --- | --- | --- | --- |
| BaikalG2 | *Cyanobium*/*Synechococcus* | 1166823 | 63.8 | NM1 | Unknown |
| 8F6 | *Cyanobium*/*Synechococcus* | 2510697 | 65.81 | NM1 | IIB |
| GFB01 | *Cyanobium*/*Synechococcus* | 2339812 | 67.77 | NM1 | II |
| NS01 | *Cyanobium*/*Synechococcus* | 2748087 | 67.9 | NM1 | Unknown |
| PCC 7001 | *Cyanobium*/*Synechococcus* | 2834252 | 68.7 | NM1 | I |
| CB0205 | *Cyanobium*/*Synechococcus* | 2427308 | 62.97 | NM2 | II |
| CB0101 | *Cyanobium*/*Synechococcus* | 2691045 | 64.2 | NM2 | Unknown |
| LL | *Cyanobium*/*Synechococcus* | 3548884 | 68.35 | NM2 | IIB |
| CACIAM 14 | *Cyanobium*/*Synechococcus* | 3212489 | 68.56 | NM3 | I |
| PCC 6307 | *Cyanobium*/*Synechococcus* | 3342364 | 68.71 | NM3 | I |
| BO8801 | *Cyanobium*/*Synechococcus* | 3271183 | 69.15 | NM3 | I |
| WH5701 | *Cyanobium*/*Synechococcus* | 3043834 | 64.3 | Sub-C 5.2 | I |
| 1G10 | *Cyanobium*/*Synechococcus* | 3340220 | 64.56 | Sub-C 5.2 | IIB |
| MW101C3 | *Cyanobium*/*Synechococcus* | 3029138 | 66.13 | Sub-C 5.2 | I |
| Lanier | *Cyanobium*/*Synechococcus* | 1475744 | 51.4 | Sub-C 5.3 | II |
| RCC307 | *Cyanobium*/*Synechococcus* | 2224914 | 60.84 | Sub-C 5.3 | III |
| Tous | *Cyanobium*/*Synechococcus* | 2515215 | 62.6 | Sub-C 5.3 | II |
| CC9311 | Marine *Synechococcus* | 2606748 | 52.45 | I | III |
| WH 8016 | Marine *Synechococcus* | 2706690 | 54.07 | I | III |
| MED195 | Marine *Synechococcus* | 1306460 | 61.05 | I | Unknown |
| CC9605 | Marine *Synechococcus* | 2510659 | 59.22 | II | III |
| WH 8109 | Marine *Synechococcus* | 2111515 | 60.09 | II | III |
| WH 8102 | Marine *Synechococcus* | 2434428 | 59.41 | III | III |
| WH 8103 | Marine *Synechococcus* | 2429688 | 59.5 | III | III |
| RS427 | Marine *Synechococcus* | 1406456 | 61.2 | III | III |
| MED843 | Marine *Synechococcus* | 1472163 | 68.6 | III | Unknown |
| CC9902 | Marine *Synechococcus* | 2234828 | 54.16 | IV | III |
| BL107 | Marine *Synechococcus* | 2283377 | 54.24 | IV | III |
| CC9616 | Marine *Synechococcus* | 2645910 | 56.52 | IV | III |
| SAT1300 | Marine *Synechococcus* | 1082607 | 68.6 | IV | Unknown |
| KORDI-100 | Marine *Synechococcus* | 2789000 | 57.5 | UC-A | III |
| ARS6 | Marine *Synechococcus* | 2186662 | 55.5 | V/VI/VII | Unknown |
| WH 7805 | Marine *Synechococcus* | 2620367 | 57.63 | V/VI/VII | II |
| WH 7803 | Marine *Synechococcus* | 2366980 | 60.24 | V/VI/VII | III |
| RS9916 | Marine *Synechococcus* | 2664873 | 59.8 | VIII/IX | III |
| RS9917 | Marine *Synechococcus* | 2579542 | 64.46 | VIII/IX | I |
| KORDI-49 | Marine *Synechococcus* | 2585813 | 61.4 | WPC1 | III |
| KORDI-52 | Marine *Synechococcus* | 2572069 | 59.1 | WPC2 | III |
| MIT9515 | *Prochlorococcus* | 1704176 | 30.79 | HLI | NA |
| CCMP1986 | *Prochlorococcus* | 1657990 | 30.8 | HLI | NA |
| MIT9202 | *Prochlorococcus* | 1691453 | 31.08 | HLII | NA |
| MIT9215 | *Prochlorococcus* | 1738790 | 31.15 | HLII | NA |
| MIT0604 | *Prochlorococcus* | 1780061 | 31.17 | HLII | NA |
| MIT9312 | *Prochlorococcus* | 1709204 | 31.21 | HLII | NA |
| AS9601 | *Prochlorococcus* | 1669886 | 31.32 | HLII | NA |
| MIT9301 | *Prochlorococcus* | 1641879 | 31.34 | HLII | NA |
| MIT0801 | *Prochlorococcus* | 1929203 | 34.91 | LLI | NA |
| NATL1A | *Prochlorococcus* | 1864731 | 34.98 | LLI | NA |
| NATL2A | *Prochlorococcus* | 1842899 | 35.12 | LLI | NA |
| CCMP1375 | *Prochlorococcus* | 1751080 | 36.44 | LLII/LLIII | NA |
| MIT9211 | *Prochlorococcus* | 1688963 | 38.01 | LLII/LLIII | NA |
| MIT9303 | *Prochlorococcus* | 2682675 | 50.01 | LLIV | NA |
| MIT9313 | *Prochlorococcus* | 2410873 | 50.74 | LLIV | NA |
| 142 | Sponge symbionts | 2271413 | 58.7 | Symbionts | B |
| 15L | Sponge symbionts | 1445676 | 59.48 | Symbionts | B |
| SP3 | Sponge symbionts | 2129964 | 60.9 | Symbionts | B |
| SH4 | Sponge symbionts | 1658709 | 63.05 | Symbionts | B |
| PCC 7942 | *Synechococcus elongatus* | 2742269 | 55.43 | *S. elongatus* | A |
| PCC 6301 | *Synechococcus elongatus* | 2696255 | 55.48 | *S. elongatus* | A |

**Table S3**. Marginal likelihoods and Bayes factors for the hypotheses on the origin of *mpeBA*.

| Hypothesis | ln-Marginal likelihood | ln-Bayes factor *vs* H_3_ |
| --- | --- | --- |
| H_1_: Ancient duplication | -10758.90 | 4.76 |
| H_2_: Recent duplication | -10724.83 | 38.83 |
| H_3_: Lateral gene transfer | -10763.66 |  |

**Table S4**. Marginal likelihoods and Bayes factors for the hypotheses on LGT within *mpeBA*.

| Hypothesis | ln-Marginal likelihood | ln-Bayes factor *vs* H_2_ |
| --- | --- | --- |
| H_1_^*^ | -2967.16 |  |
| H_1_: Widespread LGT | -2969.50 | 172.34 |
| H_2_: Vertical descent | -3141.84 |  |
| H_3_: Vertical descent + 1LGT | -3132.39 | 9.45 |
| H_4_: Vertical descent + 4LGT | -2956.28 | 185.56 |
| H_5_: Vertical descent + 4LGT + uncertainty in *S. spongiarum* | -2949.32 | 192.52 |

**Table S5** Marginal likelihoods and Bayes factors for the pairs of hypotheses on LGT within *cpeBA*.

| Hypothesis | | ln-Marginal likelihood | ln-Bayes factor |
| --- | --- | --- | --- |
| H_1-1_: | Widespread LGT | -3294.06 | 248.73 |
| H_1-2_: | Vertical descent | -3542.79 |  |
| H_2-1_: | *S. sp.* CB0205 and *S. sp.* WH7805 monophyletic | -3290.11 | 9.55 |
| H_2-2_: | *S. sp.* CB0205 and *S. sp.* WH7805 not monophyletic | -3299.66 |  |
| H_3-1_: | From *S. sp* CB0205 to *S. sp.* WH7805 | -3290.60 | 31.95 |
| H_3-2_: | From *S. sp* WH7805 to *S. sp.* CB0205 | -3322.55 |  |
| H_4-1_: | *S. sp.* RCC307 inside Type 3 | -3280.16 | 33.63 |
| H_4-2_: | *S. sp.* RCC307 outside Type 3 | -3313.79 |  |

**
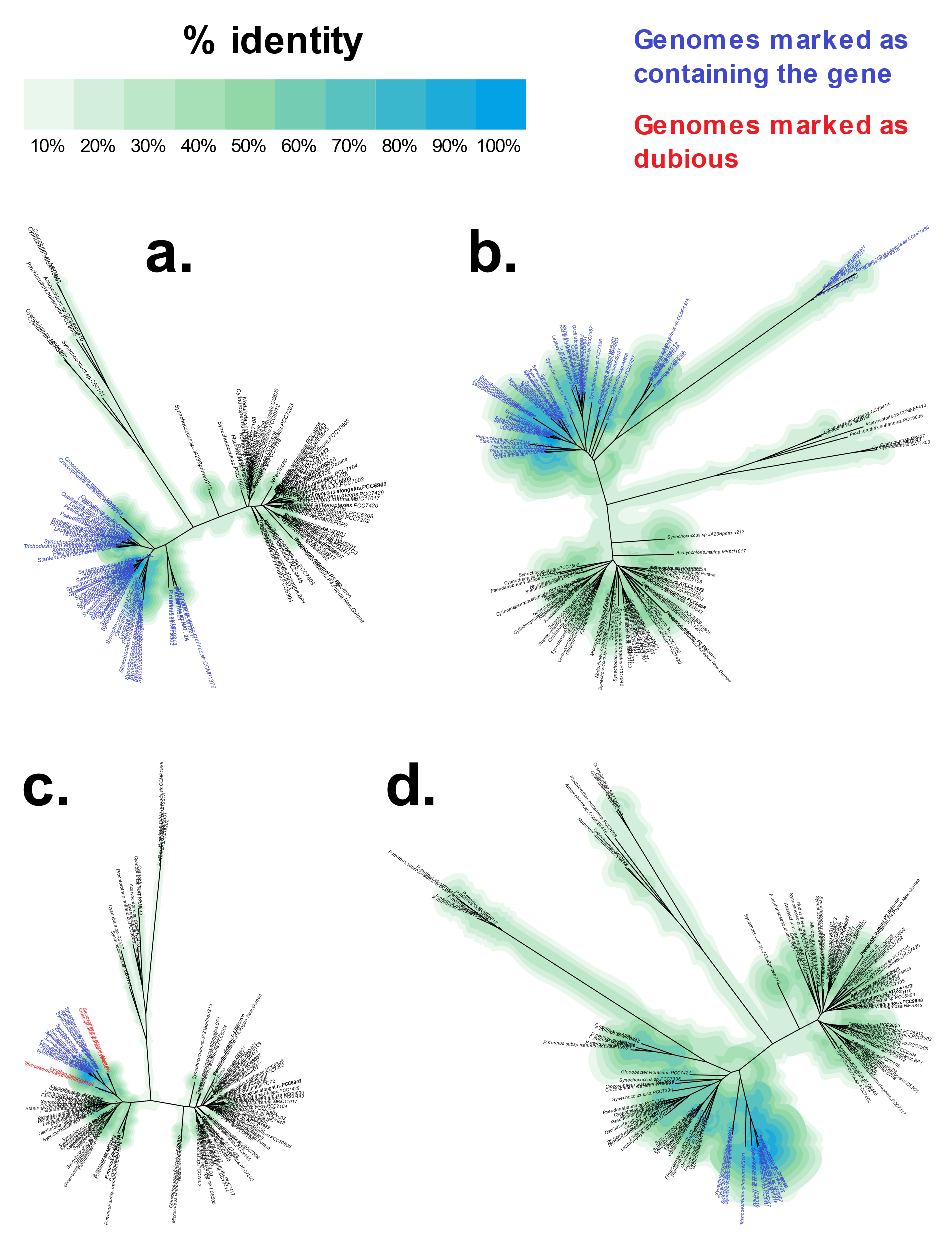
**

**Figure S1.** Neighbour-joining topologies illustrating *cpeBA* and *mpeBA* orthologs. **a.** *cpeA*; **b.** *cpeB*; **c.** *mpeA*; **d.** *mpeB*

**
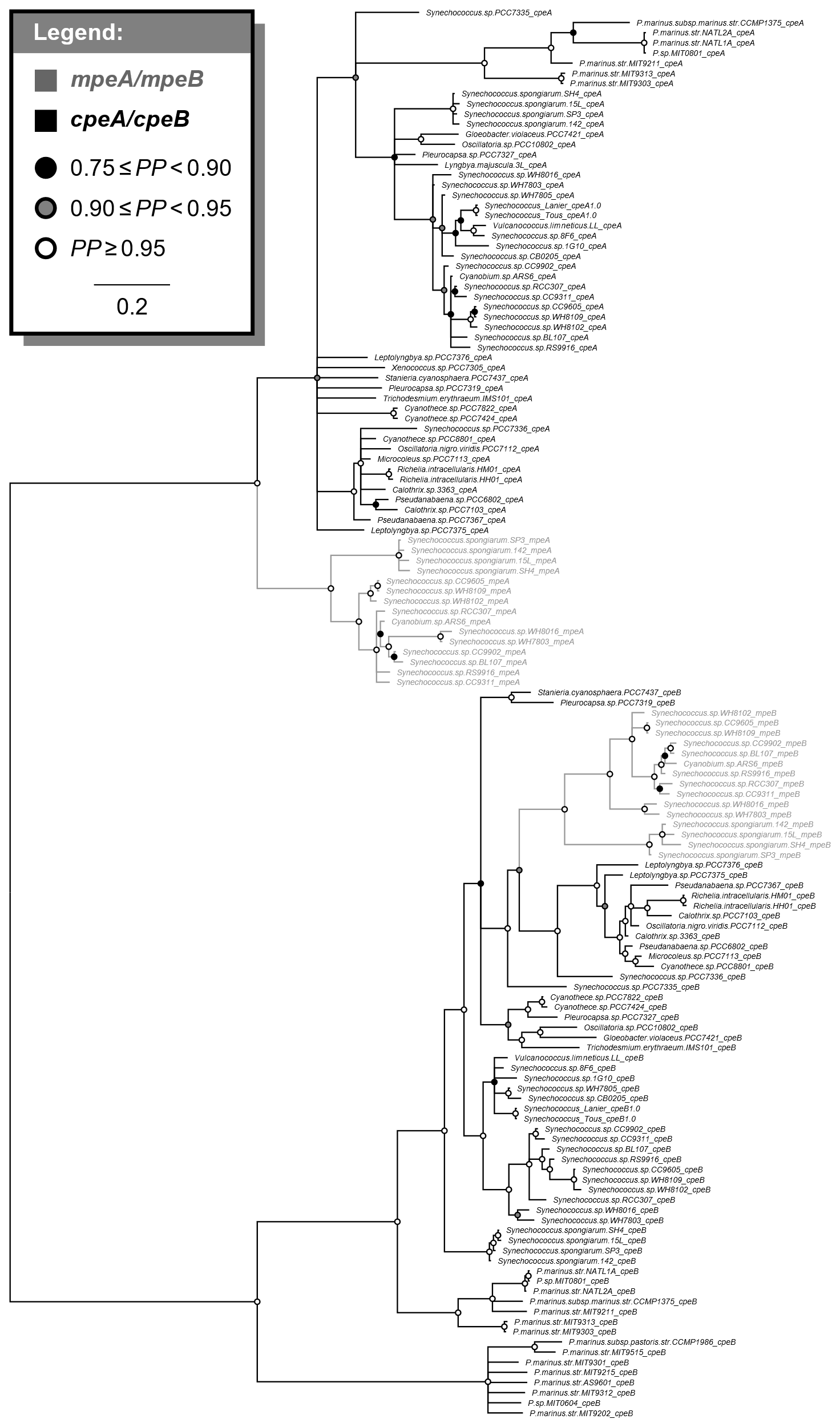
**

**Figure S2.** Bayesian phylogenetic tree of the *cpeA*, *cpeB*, *mpeA* and *mpeB* genes. *mpeA* and *mpeB* sequences are highlighted in grey. PP: Posterior Probability. Branches with PP < 0.75 were collapsed.

**
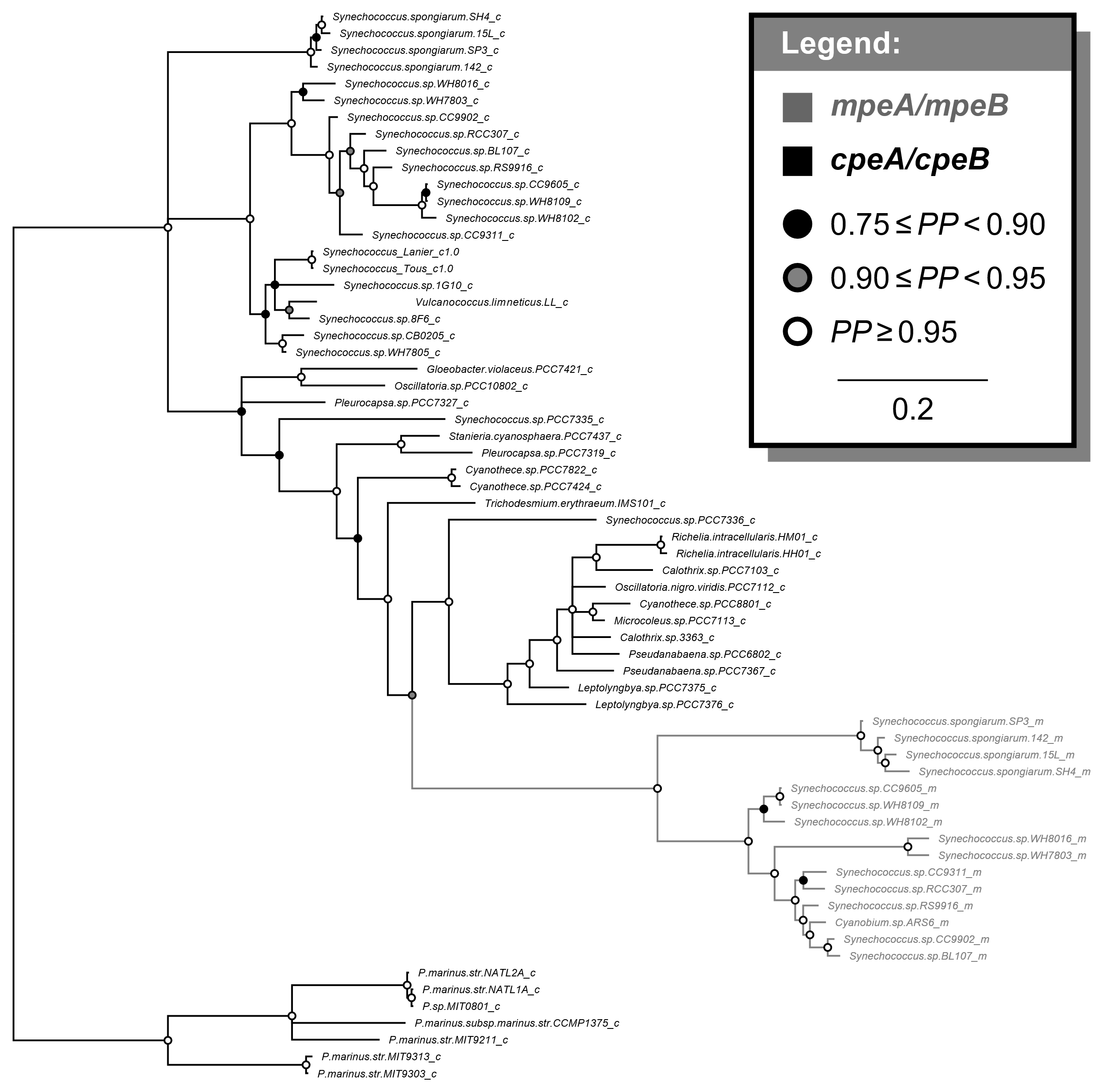
**

**Figure S3.** Bayesian phylogenetic tree of the *cpeBA* and *mpeBA* concatenated genes. *mpeBA* sequences are highlighted in grey. PP: Posterior Probability. Branches with PP < 0.75 were collapsed.

**
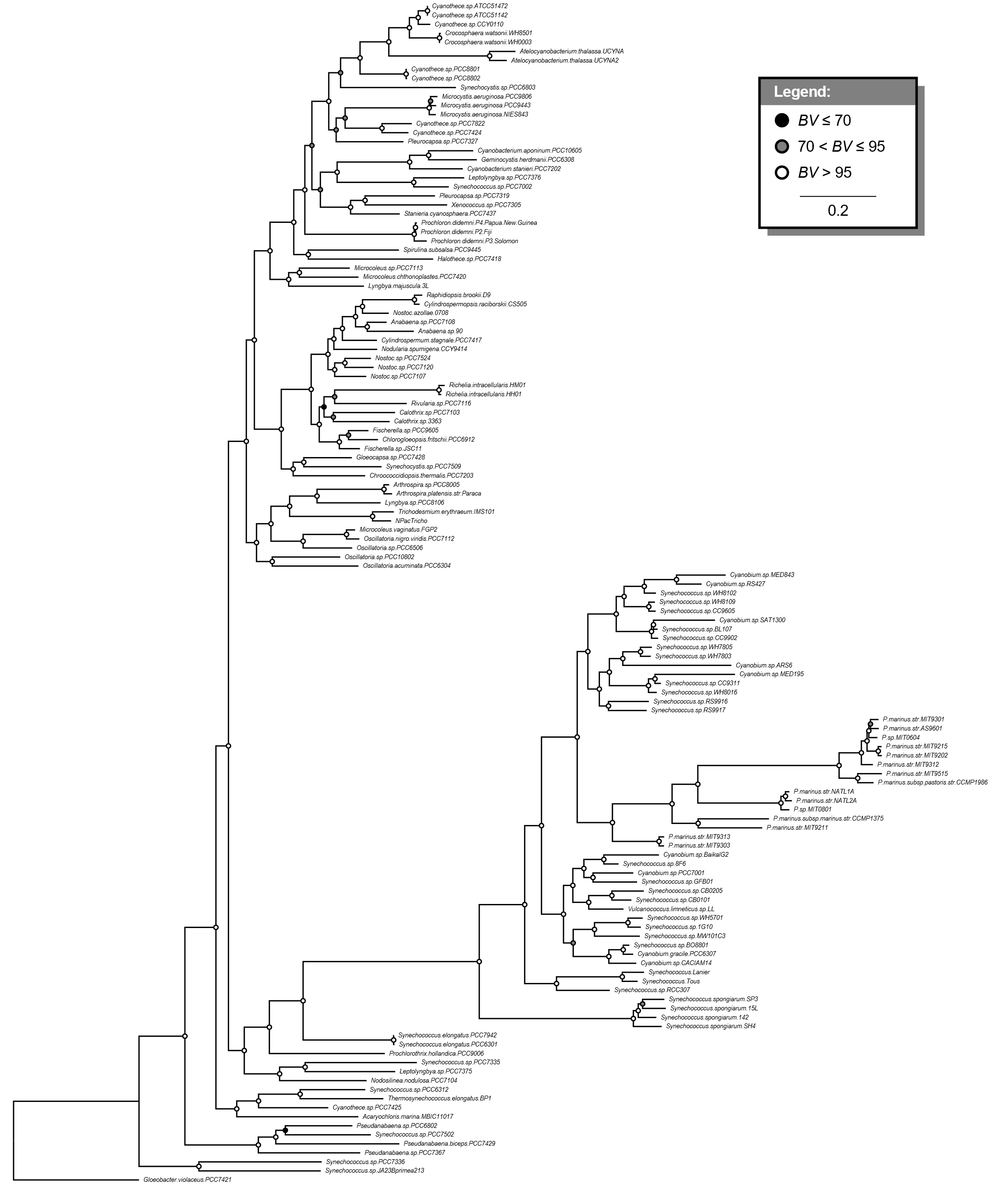
**

**Figure S4.** Phylogeny of 131 cyanobacteria genomes based on 136 proteins.


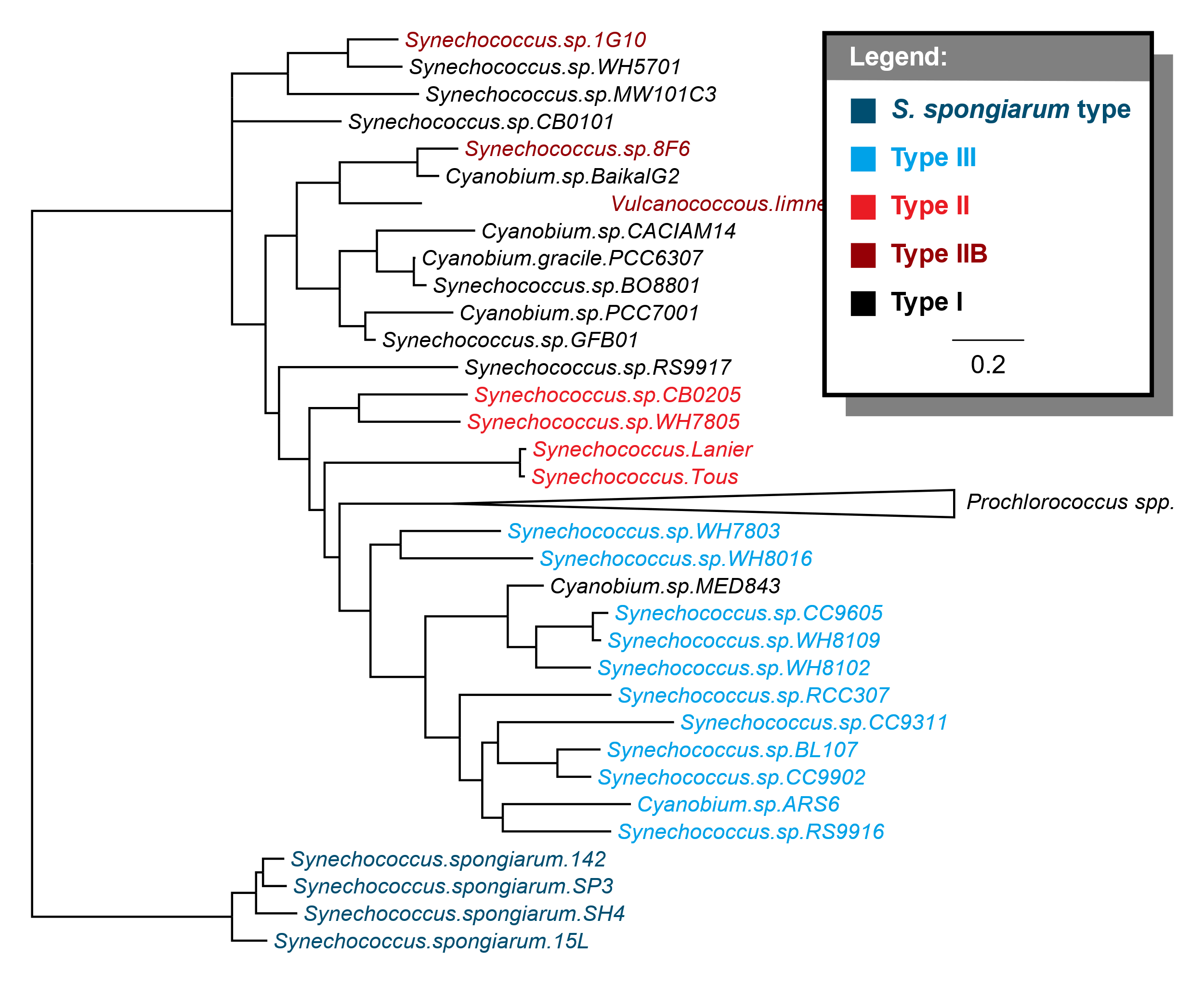


**Figure S5.** Bayesian phylogenetic tree of the phycobilisome cluster. Branches with posterior probability (PP= < 0.75 were collapsed. All remaining branches have PP > 0.95.


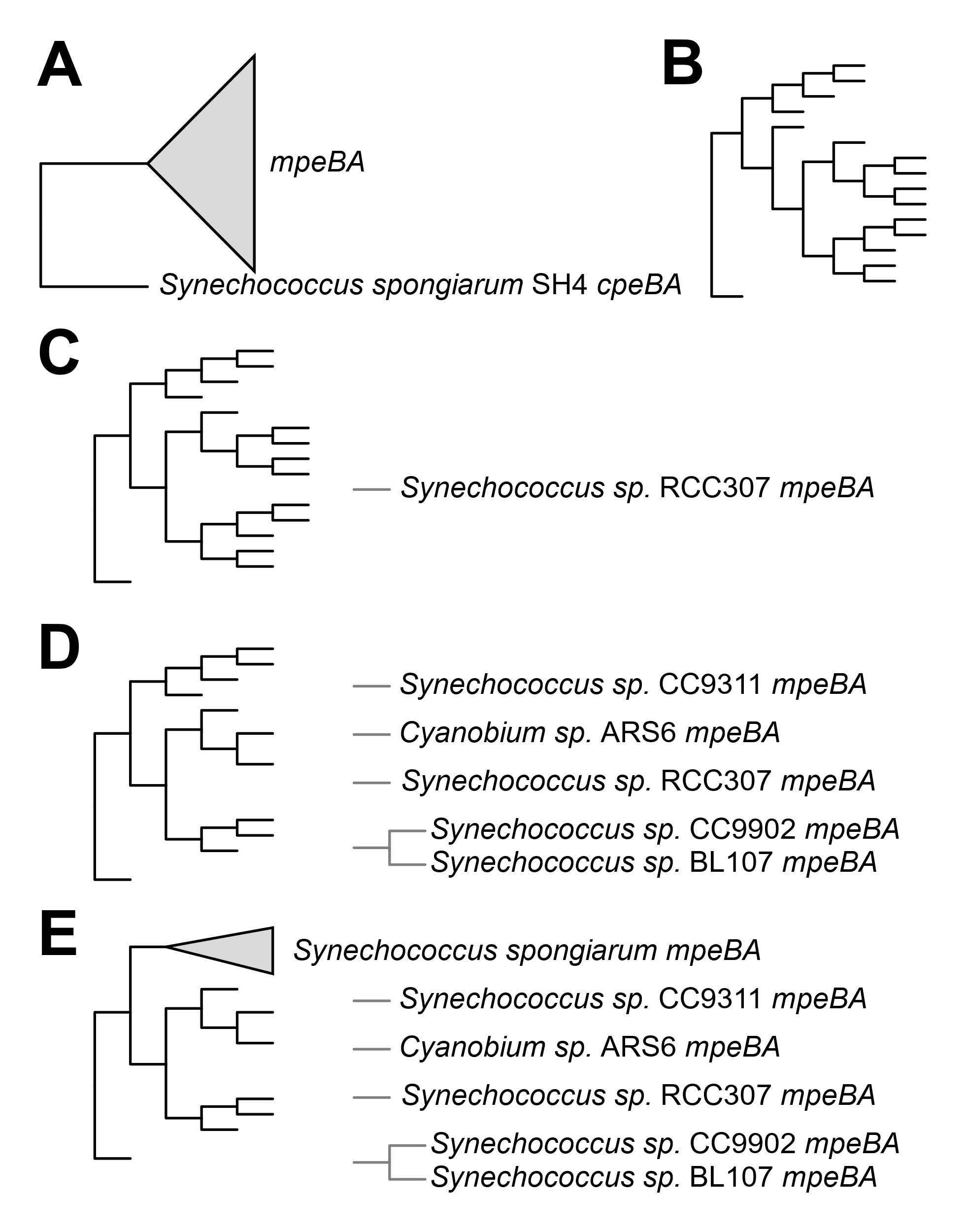


**Figure S6.** Prior tree constraints for the hypotheses for the evolution of *mpeBA* genes. **A**: hypothesis 1 – widespread LGT. **B**: hypothesis 2 – exclusive vertical descent (with some losses). **C**: hypothesis 3 – vertical descent with losses and a lateral gene transfer (LGT) event involving *Synechococcus sp.* RCC307 (i.e. this strain is left “floating” in the prior, and can attach anywhere in the tree). **D**: hypothesis 4 – vertical descent with losses and 4 LGT events involving *Synechococcus sp.* RCC307, *Synechococcus sp.* 9311, *Synechococcus sp.* CC9902, *Synechococcus sp.* BL107 and *Cyanobium sp.* ARS6 (these species are left floating, but *Synechococcus sp.* CC9902 and *Synechococcus sp.* BL107 are constrained to form a monophyletic group). **E**: vertical descent with losses, 4 LGT events (same as in **D**) and uncertainty in *S. spongiarum* relationships (the *S. spongiarum* are constrained to form a monophyletic group, but the relationships between them can take on any shape).


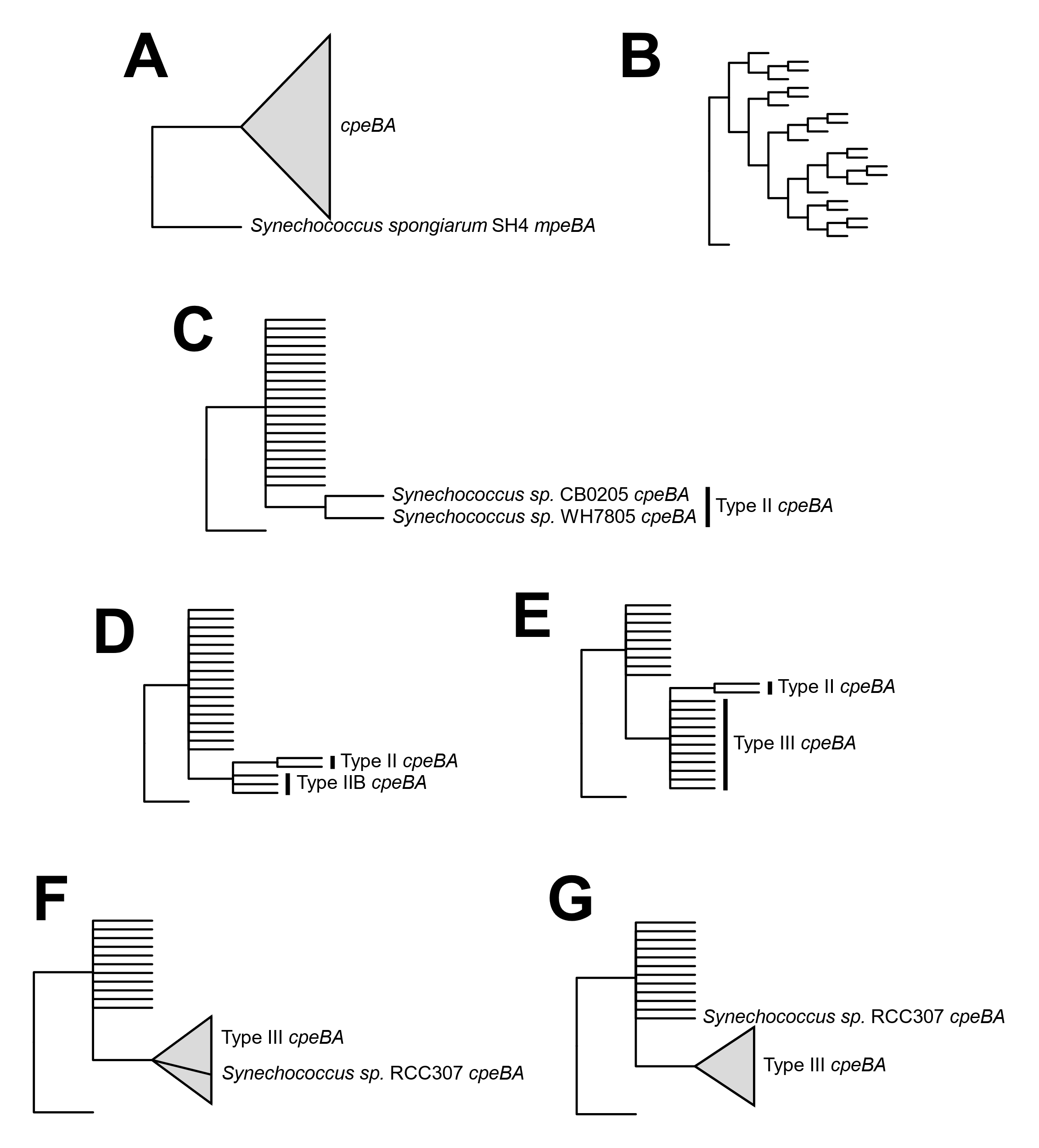


**Figure S7.** Prior tree constraints for the hypotheses for the evolution of *cpeBA* genes. **A**,**B**: H_1-1_ – widespread LGT (**A**) *vs* H_1-2_ – pure vertical descent (**B**). **C**: H_2-1_ – Type II *cpeBA* (i.e. the ones from *Synechococcus sp.* 0205 and *Synechococcus sp.* WH7805) monophyletic (**C**) *vs* H_2-2_ – non-monophyletic (tree not shown). **D,E**: H_3-1_ – LGT from an ancestor of *Synechococcus sp.* CB0205 to an ancestor of *Synechococcus sp.* WH7805 (**D**) *vs* H_3-2_ – LGT from an ancestor of *Synechococcus sp.* WH7805 to an ancestor of *Synechococcus sp.* CB0205 (**E**). **F,G**: H_4-1_ – *cpeBA* of *Synechococcus sp.* RCC307 nested within Type 3 pigment cluster species (**F**) *vs* H_4-2_ – Type 3 pigment cluster species making up a monophyletic cluster without *Synechococcus sp.* RCC307 (**G**).

**Literature**

Felsenstein, F. . *Inferring Phylogenies.* Sunderland, Massachussets: Sinauer Associates Inc, 2004.

Kass, R. E.; Raftery A. E. . "Bayes Factors." *Journal of the American Statistical Association* 90, no. 430 (2012): 773-95.

Ronquist, F., M. Teslenko, P. van der Mark, D. L. Ayres, A. Darling, S. Hohna, B. Larget*, et al.* "Mrbayes 3.2: Efficient Bayesian Phylogenetic Inference and Model Choice across a Large Model Space." *Syst Biol* 61, no. 3 (May 2012): 539-42.

Wolfram|Alpha. "Wolfram Alpha Llc." <http://www.wolframalpha.com>.

Xie, W., P. O. Lewis, Y. Fan, L. Kuo, and M. H. Chen. "Improving Marginal Likelihood Estimation for Bayesian Phylogenetic Model Selection." *Syst Biol* 60, no. 2 (Mar 2011): 150-60.
